# Removal of CdS-QDs pollutant from wastewater by interconnected Ca/Al layered double hydroxides with hierarchical mesoporous calcite and chitosan hydrogel

**DOI:** 10.1038/s41598-026-43797-x

**Published:** 2026-04-02

**Authors:** Mohamed E. Mahmoud, Mohamed F. Amira, Enass A. I. Saleh, Hany Abdel-Aal

**Affiliations:** Faculty of Sciences, Chemistry Department, Alexandria University, Alexandria, Egypt

**Keywords:** CdS-QDs remediation, nanobiocomposite, Hierarchical mesoporous calcite, CaAl-layered double hydroxide, Chitosan hydrogel, Thermodynamic and kinetic studies, Chemistry, Environmental sciences, Materials science, Nanoscience and technology

## Abstract

Cadmium sulfide quantum dots (CdS-QDs) have found extensive use in optoelectronic and biomedical technologies but their high solubility and instability in aqueous environments were identified to enforce significant environmental and health concerns due to leaching of Cd²⁺ ions. The aim of this investigated is devoted to achieve effective CdS-QDs removal from aquatic systems via assembly of a novel nanobiocomposite of hierarchical mesoporous calcite embedded in a chitosan hydrogel reinforced into CaAl layered double hydroxides for the formation of HMC@CH@CaAl-LDH. Structural and compositional analyses (FT-IR, XRD, SEM/TEM, EDX, BET, and TGA) confirmed the successful integration of mesoporous calcite and CaAl-LDH within the chitosan matrix, yielding a thermally stable, mesoporous hybrid with abundant functional groups and high surface reactivity. Adsorption experiments revealed a maximum CdS-QDs removal efficiency of 97.4% (pH 7) with a rapid equilibration at 35 min. Kinetic modeling showed excellent agreement to pseudo-second-order model, referring to chemisorption mechanism which is supported by Elovich and intraparticle diffusion assessments. Isotherm analysis demonstrated a good conformity to the Langmuir model (R² = 0.995), providing monolayer adsorption of CdS-QDs on homogeneous sites of HMC@CH@CaAl-LDH, while Freundlich, D-R, Temkin, and Flory–Huggins models highlighted surface heterogeneity and spontaneous thermodynamic feasibility (ΔG° = − 7.50 kJ/mol). Thermodynamic parameters verified the spontaneous occurrence of the process with an endothermic character. The HMC@CH@CaAl-LDH nanobiocomposite also showed durable reusability via sustaining greater than 70% of its initial efficiency after four cycles. HMC@CH@CaAl-LDH achieved > 90% removal of CdS-QDs from wastewater matrices. These results demonstrate that the evaluated HMC@CH@CaAl-LDH is a robust, sustainable, and superior performance in CdS-QDs remediation from contaminated water.

**Supplementary Information:**

The online version contains supplementary material available at 10.1038/s41598-026-43797-x.

## Introduction

Cadmium sulfide quantum dots (CdS-QDs) are semiconductor nanomaterials with sizes typically in the range of 1–10 nm and exhibit unique size-dependent optical and electronic properties arising from quantum confinement effects^1,2^. These characteristics have enabled their widespread application in bioimaging, sensing, photocatalysis, and photovoltaic devices^3,4^. Despite their technological relevance, increasing evidence indicates that CdS-QDs pose significant environmental and health risks, particularly when released into aquatic systems^5,6^. The toxicity of CdS-QDs is primarily associated with their cadmium-based core and limited chemical stability. Under environmental or physiological conditions, CdS-QDs can undergo photodegradation, resulting in the release of Cd^2+^ ions^7–9^. Cadmium is a well-known toxic heavy metal linked to carcinogenicity, nephrotoxicity, hepatotoxicity, and reproductive disorders^10^. In addition to ion release, CdS-QDs can induce reactive oxygen species (ROS) generation and interact nonspecifically with biological macromolecules, leading to oxidative stress and cellular dysfunction^11^. The extent of these toxic effects depends on several physicochemical parameters, including particle size, surface chemistry, solubility, concentration, and exposure duration^12^. Accordingly, Once introduced into aquatic environments, CdS-QDs can coexist with dissolved Cd^2+^ ions, creating complex contamination scenarios that are difficult to treat using conventional water treatment processes^13,14^. Consequently, the development of effective and sustainable remediation strategies for CdS-QDs has become an urgent environmental priority.

However, most recently published studies involving QD-based materials in water treatment have primarily focused on their application as catalysts or adsorbents. To the best of our knowledge, there are no or limited studies, if any, have addressed the direct removal of CdS-QDs from aquatic systems. Accordingly, the novelty of the present work is centered on the development and investigation of a designedsustainable hybrid nanobiocomposite for the efficient remediation of CdS-QDs from aqueous environments.

Nanobiocomposites are biopolymer-based nanocomposites, have gained increasing attention as environmentally friendly adsorbents^15^. Natural biopolymers such as chitosan offer biodegradability, low toxicity, and abundant functional groups capable of binding metal-containing pollutants^16,17^. The incorporation of inorganic functional materials such as Layered double hydroxides (LDHs) and/or Hierarchical mesoporous calcite (HMC), into biopolymer matrices has been shown to significantly enhance adsorption performance and structural stability^18–20^. LDHs are hydrotalcite-like anionic clays characterized by positively charged metal hydroxide layers and exchangeable interlayer anions^21^. The formula of LDH is typically represented as [M^2 +^ _1−x_M^3+^_x_ (OH)_2_]^x+^(A^n−^)_x/n_·yH_2_O, where M^3+^ and M^2+^ denote to trivalent and divalent metal ions, respectively. A^n−^ and y refer to interlayer anions and water molecules in the LDH layers^22^.When embedded within biopolymer matrices, LDHs act as multifunctional fillers that improve porosity, surface area, and adsorption efficiency through synergistic interactions with polymer functional groups^23–26^. On the other hand, HMC is a structurally engineered form of calcium carbonate that has recently emerged as a promising adsorbent due to its interconnected micro-, meso-, and macroporous architecture^27^. This hierarchical structure provides high surface area, enhanced mass transfer, and excellent accessibility to adsorption sites, making HMC especially suitable for capturing nano-sized pollutants^28–30^.

Therefore, the current work is aimed to integrate HMC with biopolymer and LDHs through the synthesis of a hierarchical mesoporous calcite-embedded-chitosan hydrogel and reinforced with CaAl-layered double hydroxide (HMC@CH@CaAl-LDH) as a robust hybrid nanobiocomposite with improved CdS-QDs adsorption capacity and reusability. Comprehensive structural, morphological, and physicochemical characterizations of the prepared nanobiocomposite are performed using FT-IR, XRD, SEM, TEM, EDX, TGA, and BET. Furthermore, this work is also aiming to evaluate the adsorption performance of HMC@CH@CaAl-LDH for the efficient removal of CdS-QDs under various operational conditions. In addition, this study seeks to investigate the adsorption mechanism through kinetics, thermodynamics, and isotherm studies.

## Materials and experimentations

### Chemicals, materials and instruments

The employed chemicals are presented in Table S1. While, the instruments and analytical techniques utilized for structural characterization, morphological assessment, elemental composition and surface area determination of the synthesized nanobiocomposite are listed in Table S2.

### Synthesis procedures

#### CdS-QDs

Water-dispersible CdS-QDs were assembled following a tailored solvothermal approach combined with ligand-assisted stabilization, as previously reported^31^. Briefly, a solution labeled *S1*, was prepared by dissolving 7.695 g (0.01 mol) of hydrated cadmium sulfate (3.CdSO₄·8 H₂O) in 25 mL deionized water (DW). Separately, 0.780 g (0.01 mol) of sodium sulfide hydrate (Na₂S·xH₂O) was dissolved in 25 mL DW to obtain *S2* solution. Additionally, 0.372 g (0.001 mol) of disodium ethylenediaminetetraacetate (Na₂-EDTA) was dissolved in 25 mL DW to prepare *S3* solution. After that, solutions *S1* and *S2* were introduced into a 250 mL three-neck flask and mixed under nitrogen and continuous magnetic stirring at 40 °C for 1 h to initiate the formation of CdS nuclei. Subsequently, solution *S3* was added dropwise using 40 drops per min. This was maintained at 70 °C with continuous stirring for an additional 1 h, during which the solution gradually developed a clear yellow coloration, indicating the successful CdS-QDs generation. The resulting dispersion was filtered to remove any precipitated residues, and the produced yellow colloidal solution of CdS-QDs were cooled and stored below 5 °C for subsequent characterization and application experiments.

#### Hierarchical mesoporous calcite (HMC)

The HMC was synthesized based on a previously reported procedure with minor modifications^32^. In brief, a 0.5 mol/L CaCl₂ solution was obtained by dissolving the salt in 150 mL of an ethanol–DW mixture in a 1:2 volume ratio (denoted as solution A).Similarly, a 0.5 mol/L Na₂CO₃ solution was prepared using the same solvent composition (denoted as solution B). This was gradually added to solution A, while maintaining continuous stirring. Continuous stirring was continued for 24 h at 60 °C and the produced white precipitate was separated by filtration and washed with DW and ethanol to eliminate residual ions, and then air-dried at room temperature.

#### CaAl-LDH

CaAl-LDH with nitrate as the interlayer anion (CaAl-(NO₃)–LDH), at 2:1 molar ratio Ca: Al was prepared via a hydrothermal method under strongly alkaline conditions, following a modified procedure reported previously^33^. Initially, Al(NO₃)₃·9 H₂O (11.25 g) and Ca(NO₃)₂·4 H₂O (14.17 g) were dissolved in 100 mL (0.5 mol/L NaNO₃) under continuous stirring. The solution was adjusted at pH 11–12 using a dropwise added 2.0 mol/L NaOH solution. The homogeneous suspension was placed in a Teflon autoclave and treated hydrothermally at 100 °C for 36 h. Once cooled, the resulting slurry was centrifuged and washed with DW until reaching neutral pH. The purified precipitate was dried at 70 °C and subsequently milled into fine powder for further composite synthesis.

#### Synthesis of HMC@CH@CaAl-LDH nanobiocomposite

The HMC@CH@CaAl-LDH nanobiocomposite was prepared through the crosslinking of CaAl-LDH and HMC with a chitosan-based hydrogel network^34^. Initially, 2.0 g of HMC and 2.0 g of CaAl-LDH were dispersed in 100 mL DW and stirred at 60 °C for 30 min to obtain a homogeneous suspension. Separately, chitosan solution (2.0 g per 200 mL of 5% acetic acid) was added with stirring at 60 °C. The prepared chitosan solution was then slowly added dropwise to the previously prepared HMC–(CaAl-LDH) mixture under constant stirring. The formed slurry was further stirred for 4 h at 60 °C. Subsequently, 8 mL of glutaraldehyde was introduced dropwise as a crosslinking agent until complete gelation occurred. The obtained nanobiocomposite was then filtered under vacuum, washed with DW, and followed by air-drying at ambient temperature for 48 h.

### Adsorption experiments

**Fig. 1:**
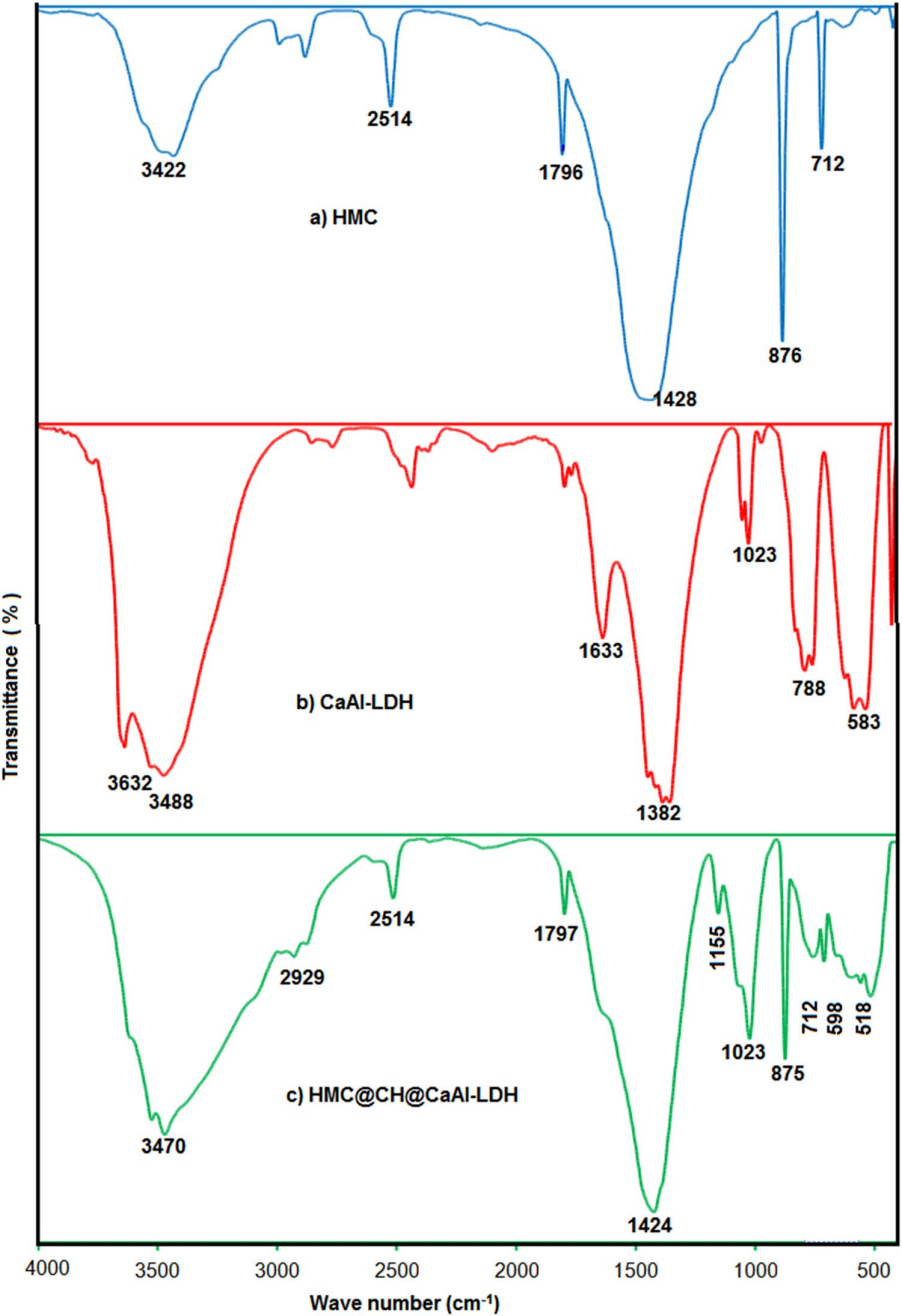
FT-IR spectra of (**a**) HMC, (**b**) CaAl-LDH, and (**c**) HMC@CH@CaAl-LDH.

Batch investigations were performed to investigate the efficiency of the as-synthesized HMC@CH@CaAl-LDH nanobiocomposite in CdS-QDs removing from water systems. In each experiment, 20 mg of the dried nanobiocomposite was delivered into a 25 mL flask containing 10 mL CdS-QDs solutions (50 and 100 mg/L). The final volume was brought to 15 mL with DW, and agitated on a mechanical shaker at 300 rpm (30 min) under ambient conditions. Following agitation, filtration was performed, and the concentration of the unadsorbed fraction of CdS-QDs in the filtrate was quantified using UV–Vis spectrophotometer. The maximum absorption wavelength (λ_max_ = 320 nm) was determined from the UV–Vis absorption spectrum (absorbance vs. wavelength) of the synthesized CdS-QDs as shown in Fig. 1S, which is consistent with a previously established standard calibration curve^35^. Equations (1) and (2) were then applied to figure out the efficiency (Removal %) and capacity (q_e_, mg/g).

1 $$\%Removal=\frac{{C}_{o}-C}{{C}_{o}}\times100$$

2 $${\mathrm{q}}_{\mathrm{e}}=\frac{\left({\mathrm{C}}_{\mathrm{o}}-\mathrm{C}\right)\mathrm{V}}{\mathrm{m}}$$

Where, C_o_ and C represent the starting and equilibrium CdS-QDs concentrations (mg/L), V (L) is the mixture volume and m (g) mass of nanobiocomposite.

The pH effect on the adsorption behavior was investigated by adjusting the pH of CdS-QDs solutions between 2.0 and 8.0 using 0.1 mol/L HCl or NaOH. The experiments were accomplished under the same batch conditions described above. For assessing the point of zero charge (pH_PZC_) of HMC@CH@CaAl-LDH, 100 mg of the material was dispersed in 50 mL (0.1 mol/L NaCl). The pH values were adjusted between 2 and 12, and the suspensions were shaken for 4 h and after contact 24 h, the pH was detected and ΔpH was used to construct a plot for pH_PZC_ determination.

To assess the impact of HMC@CH@CaAl-LDH dosage on CdS-QDs removal, adsorption experiments were conducted with varying dosages of 10, 20, 30, 40, and 50 mg, while keeping the pH fixed at 7 and maintaining 30 min time.

The role of temperature in the adsorption process was investigated by performing experiments at various temperatures (20–70 °C) under optimized conditions (pH 7 and 30 min contact time). Thermodynamic coefficients were subsequently calculated to figure out the feasibility, spontaneity, and nature of the adsorption mechanism.

The adsorption contact time influence and kinetic study were investigated by changing the shaking time from 1 to 35 min under optimized experimental conditions. Samples were withdrawn at specific intervals, and the residual CdS-QDs were analyzed to evaluate the adsorption rate and removal efficiency.

To study the influence of initial concentration and adsorption isotherms, CdS-QDs solutions with different starting concentrations (20 to 100 mg/L) were subjected to adsorption under optimized conditions (pH 7 and 30 min contact time). The obtained data were used to model adsorption equilibrium behaviors and mechanisms.

The regeneration and reusability of HMC@CH@CaAl-LDH were evaluated using successive adsorption–desorption cycles. In each cycle, 10 mL of 100 mg/L CdS-QDs mixed with 200 mg of the nanobiocomposite, diluted to 15 mL final volume, and agitated for 30 min at pH 7. After filtration, the spent HMC@CH@CaAl-LDH was regenerated by sequential treatment with 50 mL NaOH (0.1 mol/L) for 30 min, rinsing with 50 mL DW, followed by 50 mL (0.1 mol/L HCl) for 30 min, and finally washing thoroughly with DW before reuse.

The effect of ionic strength on the removal of CdS-QDs was investigated using NaCl at concentrations of 100, 200, 300, 400, and 500 mg/L. In each experiment, 20 mg of the HMC@CH@CaAl-LDH nanobiocomposite was shaken with a mixture of 5 mL CdS-QDs solution (100 mg/L) and 5 mL NaCl solution at pH 7 for 30 min. After filtration, the removal efficiency (%) of CdS-QDs was determined for each NaCl concentration.

To follow up the practical efficiency of HMC@CH@CaAl-LDH nanobiocomposite, adsorption experiments were conducted on polluted water samples with CdS-QDs (10 mg/L). Three types of water samples were tested: tap water, seawater and industrial wastewater. For each experiment, 10 mL of the polluted water sample was shaken with 50 mg of HMC@CH@CaAl-LDH for 1 h, and then filtered. In these experiments, the same water sample was treated sequentially over three consecutive runs using fresh HMC@CH@CaAl-LDH in each cycle. The reported removal efficiencies correspond to the cumulative percentage removal relative to the initial CdS-QDs concentration in the sample.

## Results and discussion

### Synthesis and structural assessment of HMC@CH@CaAl-LDH nanobiocomposite

**Scheme 1:**
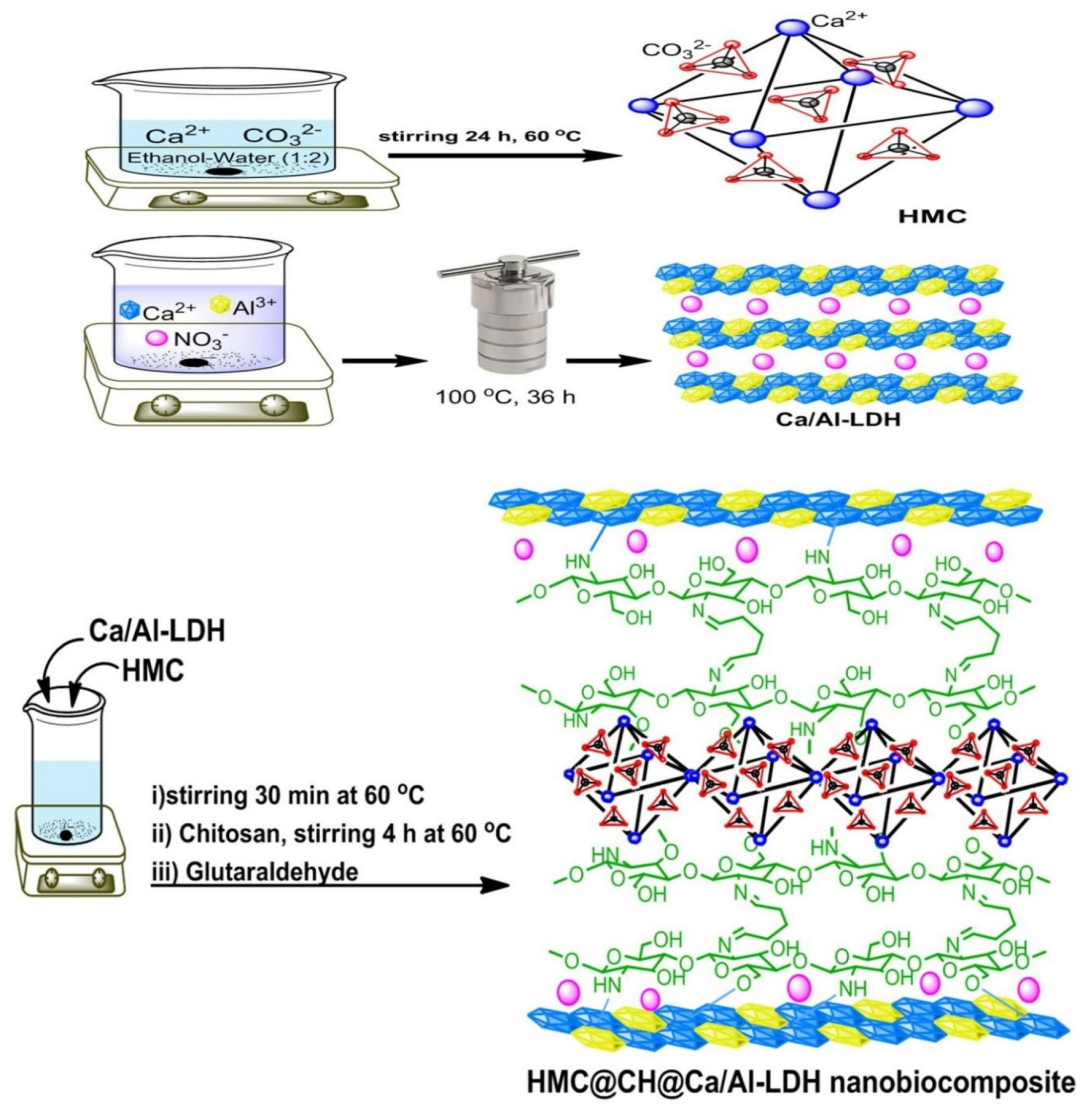
Representation of synthetic procedures ofHMC@CH@CaAl-LDH nanobiocomposite.

The HMC, CaAl-LDH, and their hybrid integration with chitosan hydrogel (CH) matrix were synthesized via a multistep process, as shown in Scheme 1. In the first step, HMC was prepared by the controlled precipitation of calcium carbonate in a water–ethanol medium, followed by self-assembly into a hierarchical mesoporous structure. In the second step, CaAl-LDH was formed by co-precipitation of calcium and aluminum precursors under alkaline conditions (pH 11–12). The reaction facilitated the nucleation and subsequent growth of LDH platelets into a well-defined hexagonal layered structure. The interlayer spacing in CaAl-LDH offered abundant hydroxyl groups and exchangeable NO_3_⁻ anions, which are crucial for strong interaction with other components and for enhancing the overall adsorption capacity. In the final step, the HMC and CaAl-LDH components were integrated within a chitosan hydrogel framework to form a robust HMC@CH@CaAl-LDH nanobiocomposite. Chitosan served as a natural crosslinking agent due to the existence of abundant –NH₂ and –OH functional groups, enabling strong chemical and hydrogen bonding between the HMC and LDH particles. The CH matrix bridges HMC microspheres and LDH nanosheets via amide linkages, hydrogen bonding, and electrostatic interactions.The structure, morphology, and surface characteristics of the HMC@CH@CaAl-LDH nanobiocomposite were examined and validated using a range of advanced characterization techniques as illustrated below.

#### FT-IR analysis

The FT-IR data were acquired to examine structural features of HMC, CaAl-LDH, and the HMC@CH@CaAl-LDH nanobiocomposite as shown in Fig. 1a, b and c respectively. The FT-IR of HMC exhibited characteristic absorption bands associated with calcite. A strong band at 1428 cm⁻¹ is owing to the asymmetric vibration of carbonate group, whilst the peaks at 876 cm⁻¹ and 712 cm⁻¹ are correlated for out-of-plane and in-plane bending of carbonate, respectively^36^. A broad band centered at 3422 cm⁻¹ denotes to surface hydroxyl groups. The FT-IR spectrum of CaAl-LDH exhibited typical features of layered double hydroxides. The intense and wide band at 3488 cm⁻¹ is designated to hydroxyl groups within the LDH layers, while a smaller band at 1633 cm⁻¹ for bending mode of interlayer water^36^. The strong peak at 1382 cm⁻¹ is referring to asymmetric stretching of intercalated nitrate anions. Additional peaks in the lower wavenumber region, including those at 583 and 788 cm⁻¹, correspond to Ca–O and Al–O lattice vibrations^37^. The FT-IR of HMC@CH@CaAl-LDH spectrum shows a broad band at 3470 cm⁻¹ attributes to the combined stretching vibrational modes of O–H and N–H groups from both chitosan and LDH hydroxyl groups. The bands at 2982 and 2929 cm⁻¹ are correlated to C–H stretching vibrations from the chitosan backbone^38^. The carbonate stretching band at 1424 cm⁻¹ from HMC remained prominent, indicating that the calcite phase was preserved in the composite. Additionally, new bands at 1155 cm⁻¹ and 1023 cm⁻¹ for C–O–C stretching vibrations from chitosan were identified^39^. Peaks in the region 875–518 cm⁻¹ represent combined contributions from carbonate bending and metal–oxygen lattice vibrations^40^. The comparative FT-IR analysis clearly demonstrates the successful formation of the HMC@CH@CaAl-LDH nanobiocomposite.

#### XRD analysis

**Fig. 2:**
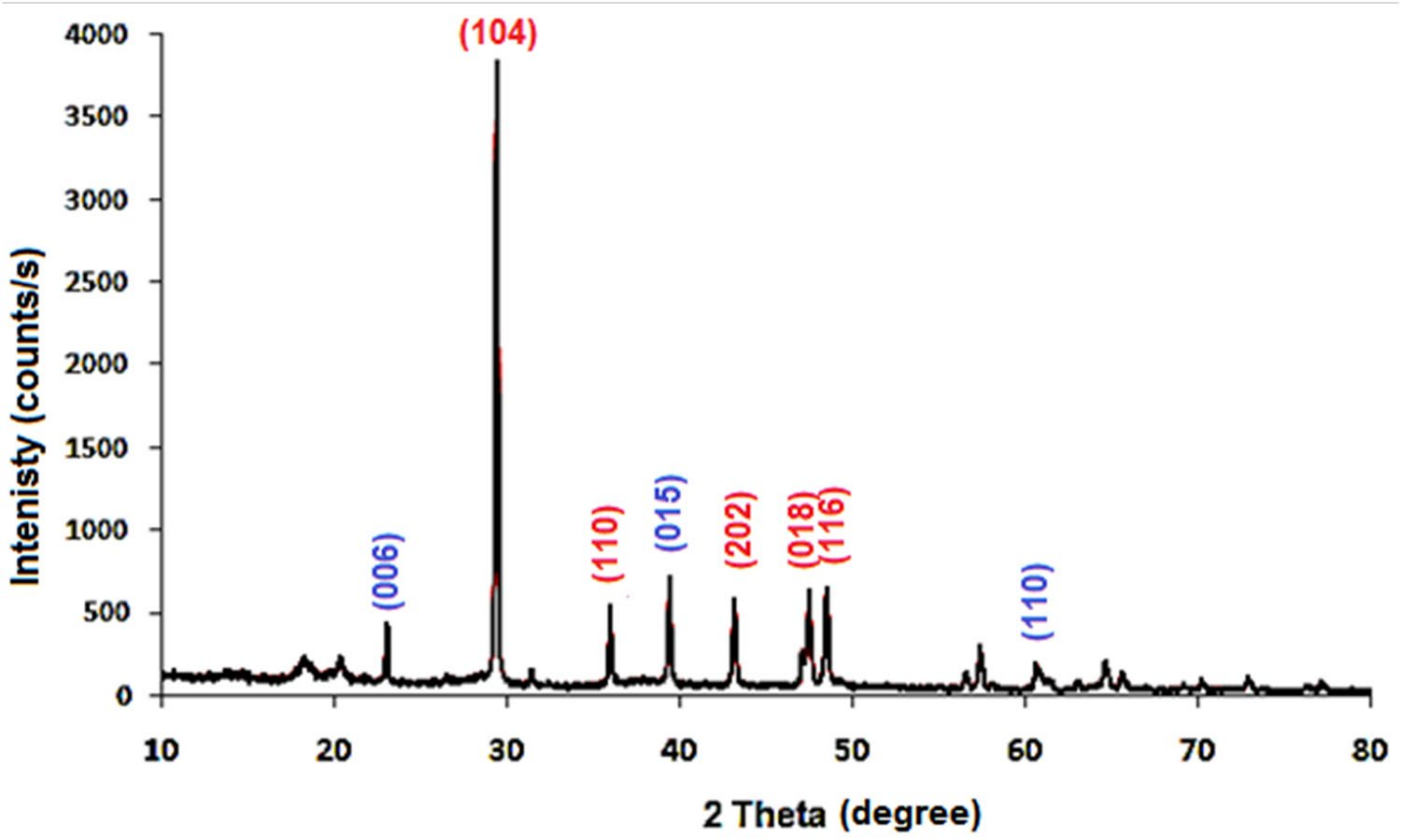
XRD pattern of HMC@CH@CaAl-LDH.

The crystal structure of HMC@CH@CaAl-LDH nanobiocomposite was investigated using XRD, and the obtained pattern is displayed in Fig. 2. The diffractogram exhibited a series of well-defined and sharp reflections, indicating the crystalline nature of the composite. The most intense reflection at 2θ ≈ 29.5°is assigned to calcite plane (104)^41^. The rhombohedral calcite structure of HMC is characterized by the sharp peaks observed at 2θ ≈ 29.5° (104), 36.0° (110), 43.2° (202), 47.5° (018), and 48.5° (116)^42^. In addition, the existence of CaAl-LDH was confirmed by diffraction peak appeared at 2θ ≈ 23.3° (006), 39.1° (015), and 60.4° (110). These reflections are consistent with the hydrotalcite-like layered structure, denoting to the ordered stacking of LDH layers along the c-axis^43^. The broad background hump indicates the presence of amorphous chitosan within the nanobiocomposite matrix due to its semi-crystalline nature^44^.

#### Morphological and microstructural analysis

**Fig. 3:**
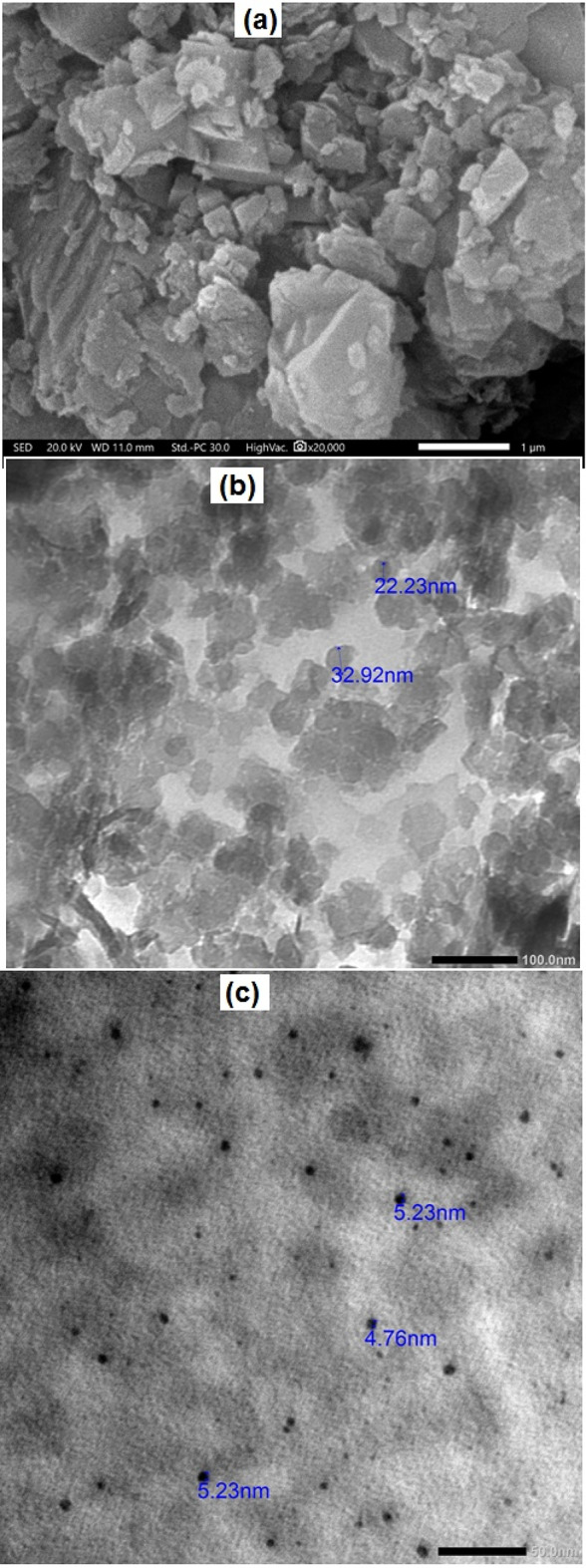
(**a**) SEM, (**b**) TEM of HMC@CH@CaAl-LDHand (**c**) TEM of CdS-QDs.

The surface morphology and internal microstructure of HMC@CH@CaAl-LDH nanobiocomposite and CdS-QDs were investigated using SEM and TEM. Figure 3a illustrates the SEM micrograph of HMC@CH@CaAl-LDH, revealing a hierarchical and irregular surface morphology composed of aggregated plate-like and block-shaped crystallites. The interconnected mesoporous structure is evident, which is attributed to the embedding of calcite within the chitosan hydrogel and reinforcement by CaAl–LDH sheets^45^. This morphology suggests that the nanobiocomposite possesses a high surface roughness and abundant active sites, which are favorable for adsorption and interaction with contaminants such as CdS-QDs. The TEM images provide further insights into the microstructure. As shown in Fig. 3b, the HMC@CH@CaAl-LDH exhibits a heterogeneous distribution of nanoparticles with sizes ranging from approximately 22.2 nm to 32.9 nm. The particles are well-dispersed within the chitosan hydrogel matrix, forming a mesoporous framework. The observed nanoscale features are consistent with the crystalline domains of calcite and LDH identified in the XRD analysis, confirming the successful integration of these phases within the hydrogel. In contrast, the TEM image of CdS-QDs (Fig. 3c) displays uniformly distributed nano-dots with much smaller particle sizes in the range of 4.7–5.2 nm. The narrow size distribution indicates that the synthesized CdS-QDs are quantum-confined, which is in good agreement with their expected optical and electronic properties. The small CdS-QDs also imply a high surface-to-volume ratio, which contributes to their strong reactivity in aqueous systems.

#### Elemental analysis and composition

**Fig. 4:**
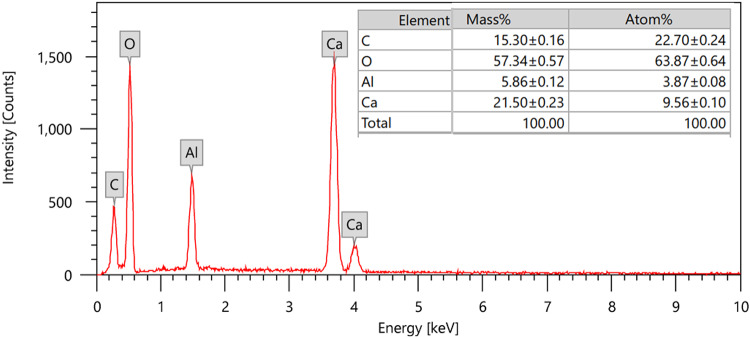
EDX analysis of HMC@CH@CaAl-LDH.

Elemental composition of the HMC@CH@CaAl-LDH nanobiocomposite was determined by EDX, as illustrated in Fig. 4. The detected elements include carbon, oxygen, calcium and aluminum as the main constituents of the composite. The strong peaks at approximately 0.3 keV, 0.5 keV, 1.5 keV, and 3.7– 4.0 keV correspond to C, O, Al, and Ca, respectively. The quantitative analysis (Fig. 4) shows mass percentages 15.30% C, 57.34% O, 5.86% Al, and 21.50% Ca. The corresponding atomic percentages are 22.70% C, 63.87% O, 3.87% Al, and 9.56% Ca. The oxygen-rich composition is consistent with hydroxylated LDH layers, interlayer water, interlayer nitrate anions and carbonate groups in calcite. The appreciable carbon content produced from both carbonate and chitosan backbones. The measured CaAl atomic ratio (2.47) reflects the averaged surface composition of the hybrid (LDH + calcite), reasonably close to the targeted LDH stoichiometry (Ca: Al = 2:1) when the additional Ca from the calcite domain is subtracted. As expected in EDX, H is not detected and N is weak/undetectable due to its low X-ray yield and overlap in the sub-keV region; nonetheless, its presence in chitosan and interlayer anions is supported by synthesis and FT-IR data. The absence of impurity peaks (such as heavy metals or other foreign elements) indicates that the synthesis procedure produced a highly pure composite showing a direct agreement with the XRD and TEM analyses and confirming the successful integration of calcite, chitosan, and CaAl-LDH phases into a single hybrid nanobiocomposite.

#### Surface analysis

**Fig. 5:**
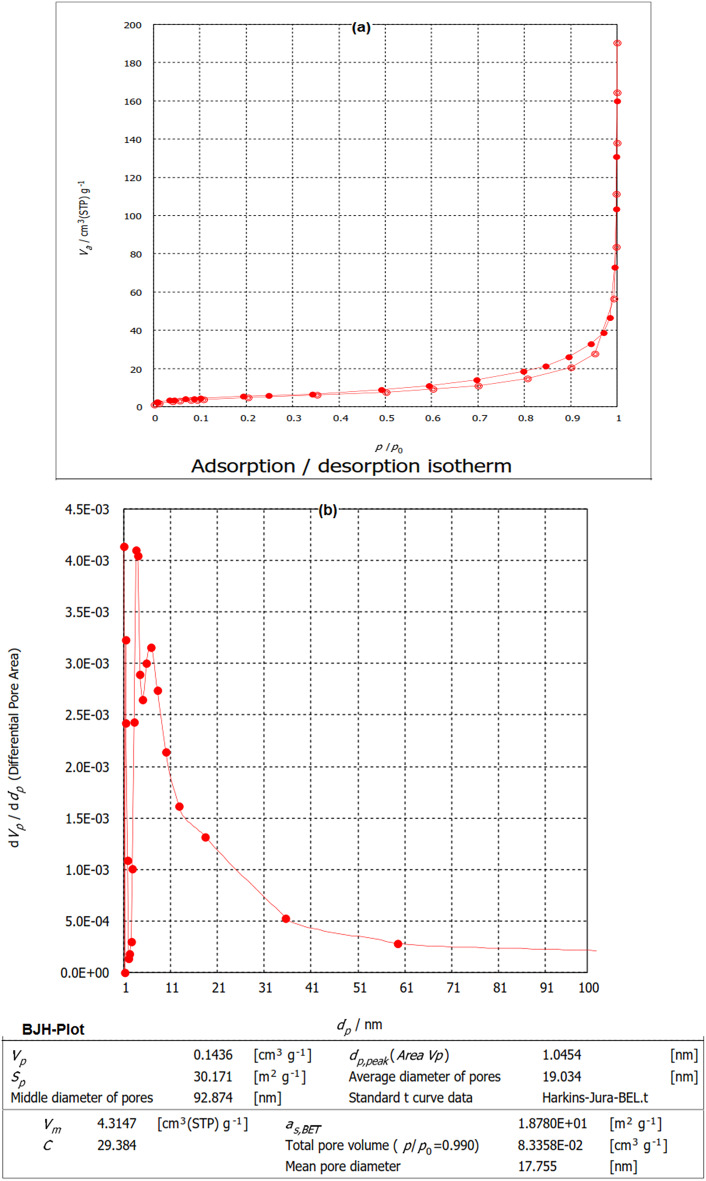
(**a**) Adsorption/desorption BET isotherm and (**b**) BJH plot of HMC@CH@CaAl-LDH.

The morphological characteristics of HMC@CH@CaAl-LDH nanobiocomposite were investigated and the results are provided in Fig. 5a. The isotherm isa IV type curve, characteristic of mesoporous structures^46^. A distinct hysteresis loop at 0.4–0.9 (P/P₀), confirms mesopores formation by the hierarchical assembly of calcite, chitosan, and CaAl-LDH within the nanobiocomposite. The resulting surface area and pore size distribution of the HMC@CH@CaAl-LDH nanobiocomposite, obtained from HJB plot (Fig. 5b), was determined to be 18.78 m^2^/g, with a calculated monolayer adsorption volume of 4.3147 cm³(STP)/g. The analysis at p/p₀ = 0.990 revealed a pore volume of 0.08335 cm³ g^− 1^ and a mean pore diameter of 17.755 nm, to further characterizing the mesoporous character of the material. Although the surface area was relatively low, the combination between hierarchical pore structure and appropriate pore size distribution enhances the diffusion and accessibility of CdS-QDs to the active sites, thereby improving the overall adsorption performance of the HMC@CH@CaAl-LDH nanobiocomposite.

#### TGA

**Fig. 6:**
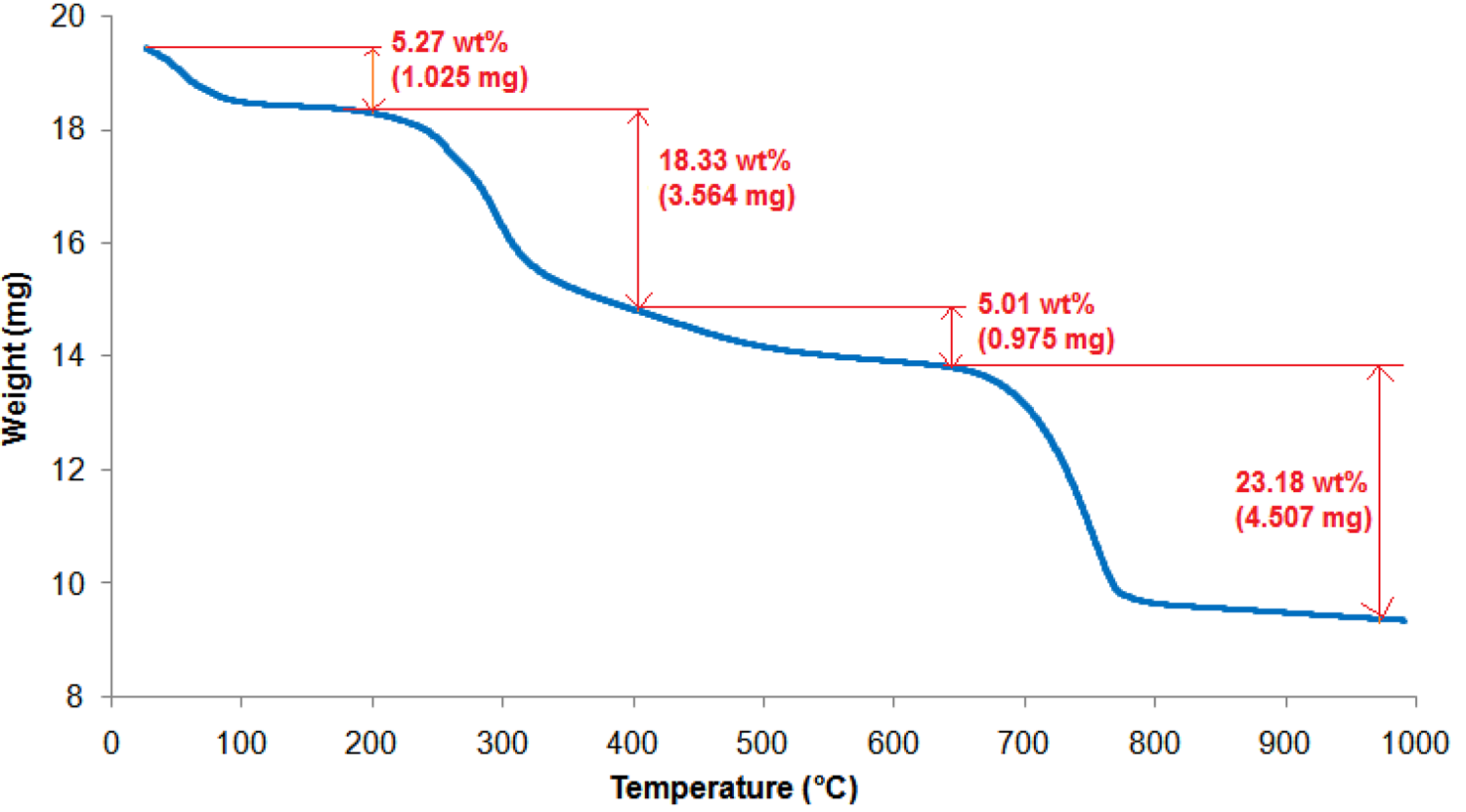
TGA of HMC@CH@CaAl-LDH.

The thermal stability of HMC@CH@CaAl-LDH nanobiocompositewas investigated in a nitrogen environment, and the resulting TGA curve is presented in Fig. 6. The thermogram shows a multi-step decomposition profile, indicative of the complex composition and hierarchical structure of the nanobiocomposite. The first weight loss at approximately 5.27% (1.025 mg) takes place below 200 °C, indicating loss of water and weakly bound moisture present within the porous structure. This initial mass loss is typical for hydrophilic materials containing hydroxyl groups, such as chitosan and layered double hydroxides^47^.The second degradation step, which accounts for 18.33% (3.564 mg) weight loss at 200 °C and 400 °C. This region is attributed mainly to possible degradation of organic materials, including the decomposition of chitosan hydrogel matrix. The presence of chitosan, a biopolymer with limited thermal stability, contributes significantly to the mass loss^48^. A third degradation step was observed between 400 °C and 650 °C, corresponding to a weight loss 5.01% (0.975 mg). This mass loss is based on complete thermal decomposition of organic residues and the partial breakdown of carbonate species associated with the calcite phase. The fourth and more substantial weight loss of 28.19% (5.482 mg) is monitored between 650 °C and 800 °C, corresponding to hydroxyl removal from LDH layers and the decomposition of interlayer NO₃⁻ anions, and complete breakdown of the inorganic calcite phase (CaCO₃) into CaO and CO₂^49^. This stage reflects the collapse of the layered architecture of the CaAl-LDH and the removal of structural hydroxyl groups. Beyond 800 °C, the curve levels off, indicating thermal stabilization of the residual metal oxide components, mainly comprising calcium and aluminum oxides. The total weight loss recorded is 51.79%, suggesting a considerable organic and volatile content within the nanobiocomposite, while the remaining mass indicates the presence of thermally stable inorganic residues.

### Adsorption analysis

#### Influence of pH

**Fig. 7:**
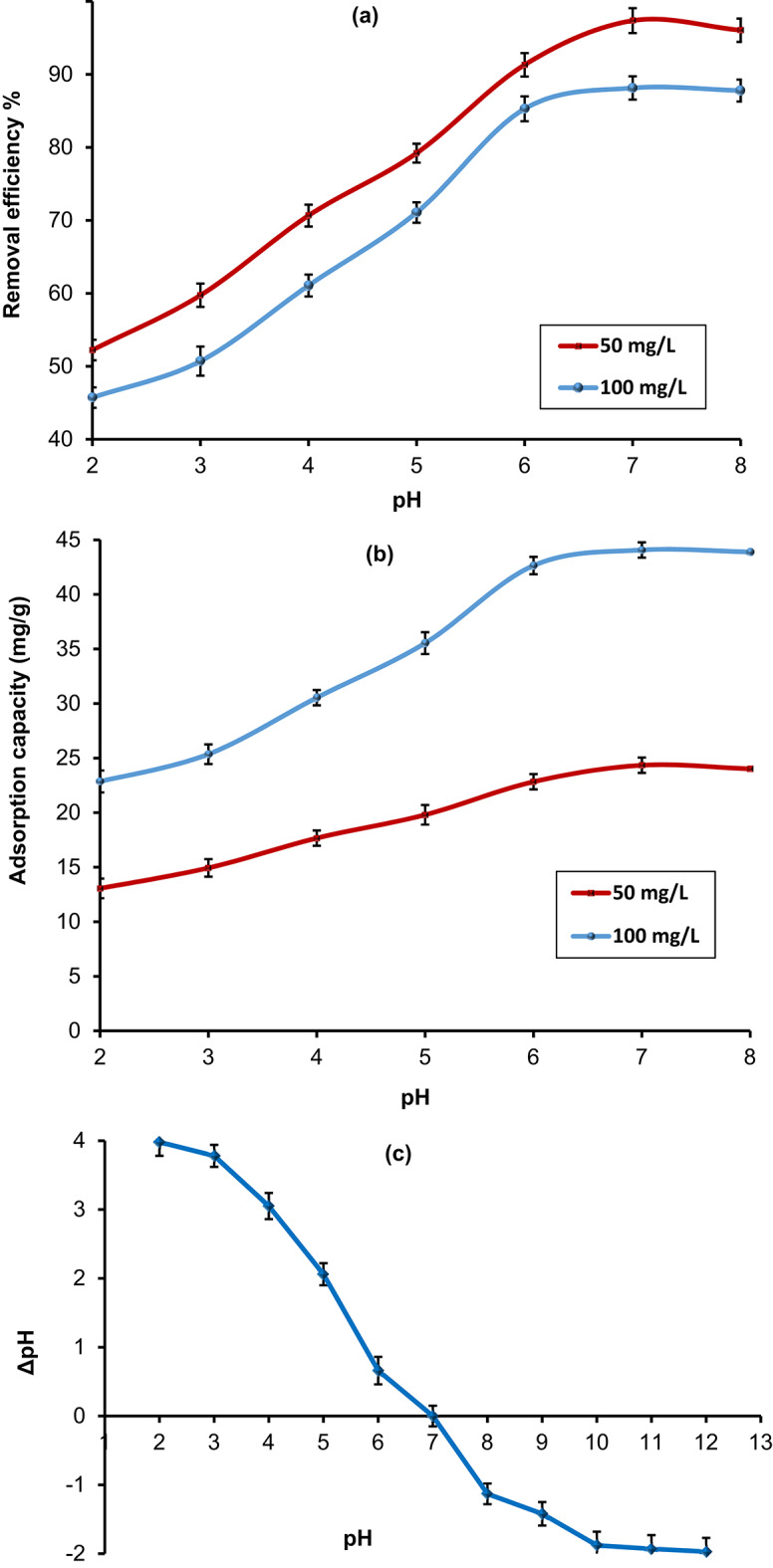
Effect of pH on (**a**) CdS-QDs removal efficiency, (**b**) adsorption capacity, and (**c**) PZC ofHMC@CH@CaAl-LDH, at 25 °C and 30 min contact time.

The solution pH is a critical factor influencing the adsorption performance of CdS-QDs on the HMC@CH@CaAl-LDH nanobiocomposite. It affects the surface charge of the adsorbent, the ionization state of functional groups, and the stability of CdS-QDs^50^. The effect of pH on removal efficiency (%) and adsorption capacity (qₑ, mg g^− 1^) was systematically investigated at two initial CdS-QDs concentrations (50 and 100 mg L^− 1^), as shown in Fig. 7a and b. Parallely, the point of zero charge (pH_pzc_) of HMC@CH@CaAl-LDH was determined to be 6.8 (Fig. 7c). This indicates that the composite surface is positively charged below pH 6.8 and negatively charged above it. The observed adsorption behavior correlates well with this pH_pzc_^51^. The optimum adsorption occurred at pH 7, with maximum removal efficiencies of 97.37% (qₑ = 24.34 mg/g) and 88.14% (qₑ = 44.07 mg/g) for 50 and 100 mg/L, respectively. Where, the surface charge is slightly negative, favoring electrostatic interactions with CdS-QDs.

At strongly acidic conditions (pH 2–4), the removal efficiency and adsorption capacity were relatively low. This is attributed to the high protonation of hydroxyl, amino, and other functional groups on the composite surface, resulting in a net positive charge that alters the interactions with CdS-QDs^52^. As the pH increased from 4 to 7, a pronounced enhancement in adsorption was observed for both concentrations. This improvement is attributed to the deprotonation of functional groups, which increases the number of available binding sites and facilitates electrostatic attraction and surface complexation between CdS-QDs and the nanobiocomposite^53^.

On the other hand, the adsorption performance of the individual components; HMC and CaAl-LDH showed lower CdS-QDs removal efficiencies (66.2% for HMC and 74.5% for CaAl-LDH at pH 7 using 50 mg/L CdS-QDs). Moreover, HMC or CaAl-LDH individually cannot withstand strongly acidic or alkaline conditions due to dissolution, degradation, or interlayer alteration. Therefore, reinforcing HMC and CaAl-LDH with chitosan hydrogel enhanced both chemical and thermal stability, resulting in improved adsorption performance for CdS-QDs.

#### Influence of HMC@CH@CaAl-LDH dosage

**Fig. 8:**
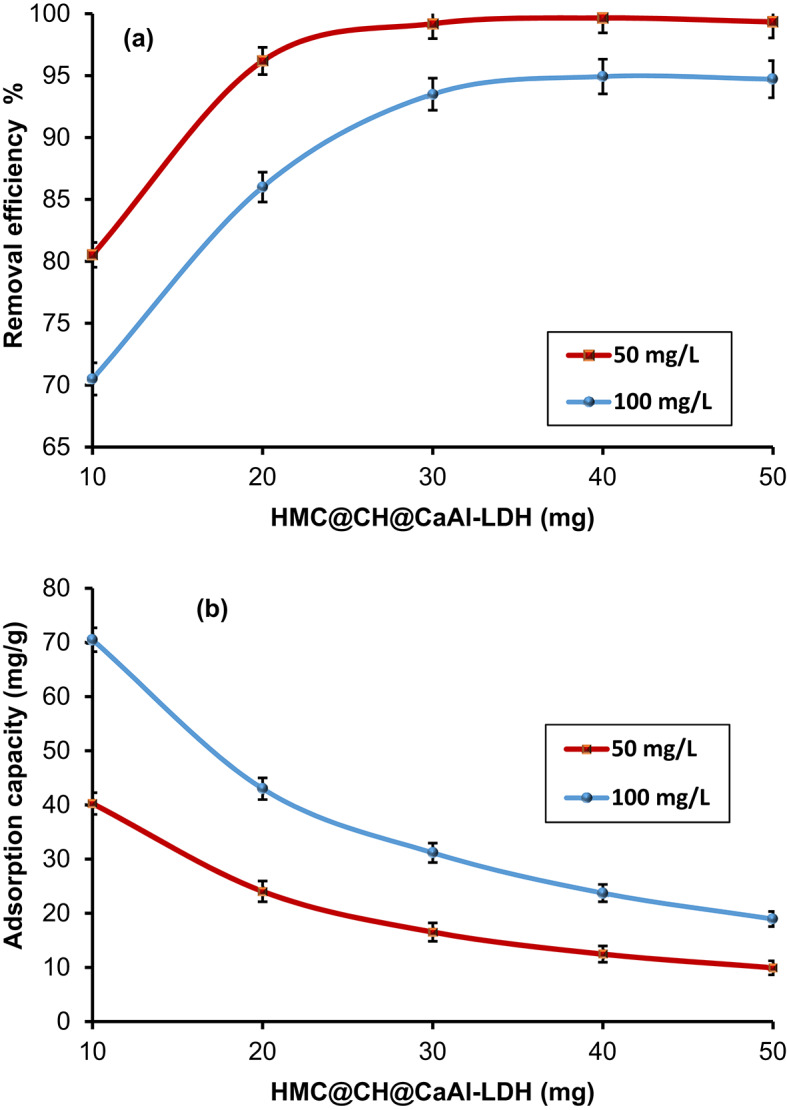
The effect of HMC@CH@CaAl-LDH mass on (**a**) removal efficiency and (**b**) adsorption capacity of CdS-QDs, at 25 °C, pH7 and 30 min contact time.

The effect of HMC@CH@CaAl-LDH mass on the removal efficiency and adsorption capacity of CdS-QDs was investigated and shown in Fig. 8a and b, respectively. The results indicate a clear positive correlation between the HMC@CH@CaAl-LDH mass and removal efficiency. At a fixed CdS-QDs concentration as 50 mg/L, the efficiency was enhanced significantly from 80.53% with 10 mg of HMC@CH@CaAl-LDH to 99.20% at 30 mg, followed by a slight change, reaching a maximum 99.34% at 50 mg. Similarly, for a higher starting concentration (100 mg/L), the removal efficiency exhibited a comparable two-stage trend, increasing from 70.50% at 10 mg to 94.71% using 50 mg. The enhanced removal efficiency at higher dosages is attributed to the increased availability of active adsorption sites provided by the composite structure^54^. In addition, the architecture significantly promoted effective diffusion and accessibility of CdS-QDs to interior adsorption sites^55^. Conversely, the adsorption capacity exhibited an inverse trend with increasing HMC@CH@CaAl-LDH dosage. This expected decrease can be explained by the definition of adsorption capacity to represent the adsorbed CdS-QDs per unit mass of the nanobiocomposite^56^.

#### Influence of temperature

**Fig. 9:**
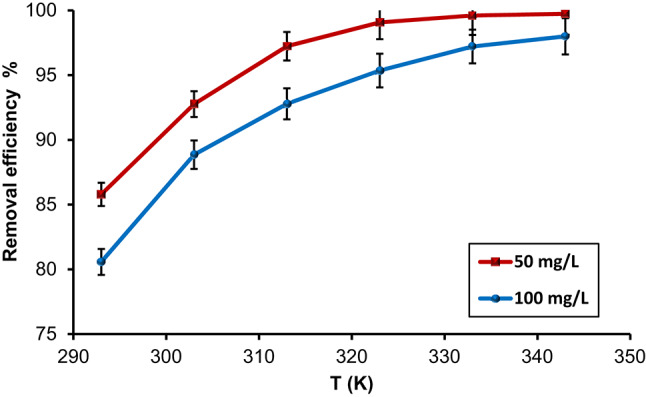
The effect of temperature on % removal efficiency of CdS-QDs by HMC@CH@CaAl-LDH, at pH7 and 30 min contact time.

The temperature impact on CdS-QDs uptake by HMC@CH@CaAl-LDH was investigated over a temperature range 293–343 K at two initial CdS-QDs concentrations (50 and 100 mg/L), as given in Fig. 9. It was observed that CdS-QDs removal was noticeably enhanced as the temperature increased, indicating an endothermic adsorption process. For 50 mg L^−1^initial concentration, the efficiency increased from 85.79% at 293 K to a maximum of 99.74% at 343 K, while at 100 mg/L, it increased from 80.57% to 98.0% across the same range of temperatures. The enhancement in adsorption at high temperatures is likely due to several contributing factors, among them the activation of additional adsorption sites, enhanced intraparticle diffusion facilitated by reduced solution viscosity, and stronger electrostatic as well as surface complexation interactions between CdS-QDs and the functional groups on HMC@CH@CaAl-LDH^57^.

The ΔG°, ΔH°, and ΔS° were determined from Fig. 2S, through applying the Van’t Hoff approach (Table 3S), and the collected data are listed in Table 1.The negative ΔG° values calculated at all tested temperatures indicate that the CdS-QDs uptake is spontaneous. For the lower initial concentration (50 mg/L), ΔG° decreased from − 2.69 kJ/mol at 293 K to − 14.96 kJ/mol at 343 K, while for 100 mg/L, it shifted from − 1.78 kJ/mol to − 9.12 kJ/mol, suggesting that the adsorption becomes increasingly favorable at higher temperatures. The positive ΔH° values at + 73.58 kJ/mol and + 41.26 kJ/mol for 50 and 100 mg/L, respectively, confirm that the process is endothermic, requiring additional energy to facilitate CdS-QDs binding^58^. Additionally, the positive ΔS° values (+ 259.61 J/mol.K for 50 mg/L and + 147.27 J/mol.K for 100 mg/L) indicate an increased degree of randomness at the solid–solution interface. This enhancement in disorder is likely attributed to water release, desorption of interlayer anions from the LDH, and detachment of surface-bound ions during adsorption, subsequent with the rearrangement of CdS-QDs within the hybrid nanobiocomposite structure^59^.

**Table 1:** Standard thermodynamic parameters of CdS-QDs adsorption by HMC@CH@CaAl-LDH.

CdS-QDs concentration	T (K)	Ln K_D_	Adsorption Thermodynamic parameters	Δ H° kJ/mol	ΔS° J/mol.K	R^2^
			Δ G° kJ/mol			
50 mg/L	293	1.10	– 2.69	73.58	259.61	0.990
	303	1.86	– 4.68			
	313	2.87	– 7.46			
	323	3.99	– 10.70			
	333	4.84	– 13.39			
	343	5.24	– 14.96			
100 mg/L	393	0.73	– 1.78	41.26	147.27	0.998
	303	1.38	– 3.48			
	313	1.86	– 4.84			
	323	2.33	– 6.25			
	333	2.86	– 7.92			
	343	3.20	– 9.12			

#### Contact time and kinetic modeling

**Fig. 10:**
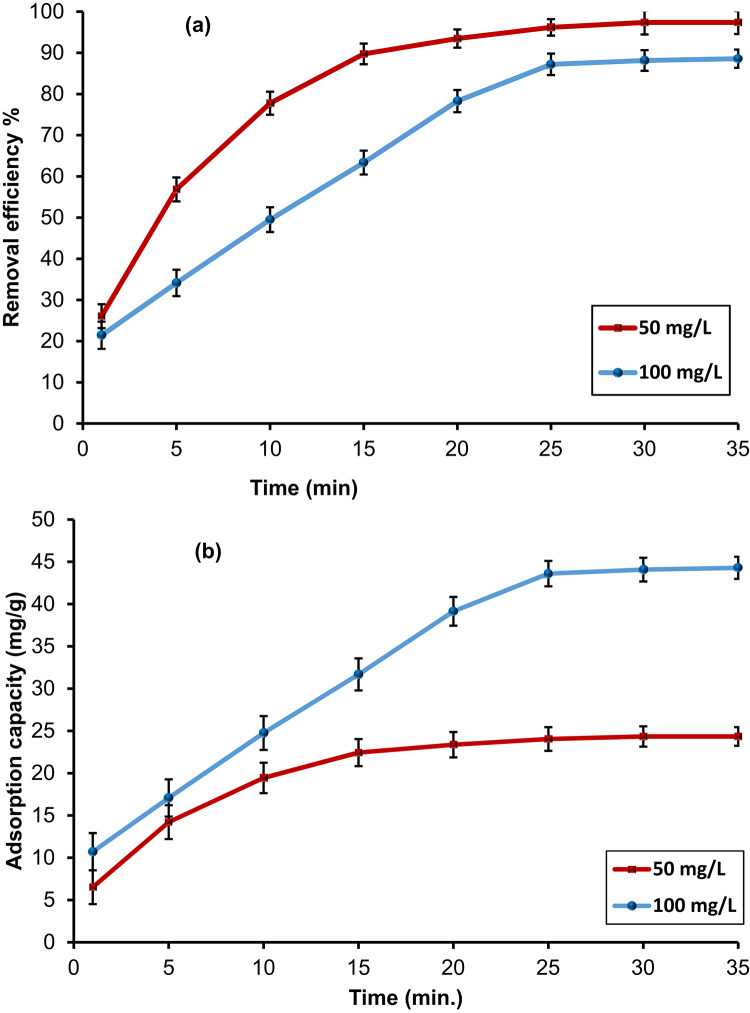
Effect of contact time on (**a**) removal efficiency and (**b**) adsorption capacity of CdS-QDs by HMC@CH@CaAl-LDH, at 25 °C andpH7.

The removal efficiency of CdS-QDs at diverse contact periods was examined to elucidate the equilibrium time as well as the adsorption kinetics. As demonstrated in Fig. 10, a sharp increase in removal efficiency was observed during the first 1–20 min, followed by a slower uptake until equilibrium was reached at 35 min. In the initial stage, the large number of accessible adsorption active sites on calcite particles and LDH nanosheets, together with the abundant functional groups (–NH₂ and –OH) of the chitosan hydrogel, allowed for rapid surface binding of CdS-QDs. The porous and hydrated nature of the chitosan matrix also enhanced diffusion of QDs toward active sites, supporting the fast initial adsorption^60^. As time progressed, the rate of adsorption decreased as a result of the progressive saturation of easily available sites. At this stage, CdS-QDs were diffused deeper into the mesoporous calcite domains and interlayer galleries of CaAl-LDH, where steric hindrance and electrostatic repulsion slowed the process. The observed plateau after 25 min indicates that equilibrium was achieved when all high-affinity sites were occupied and only less favorable sites remained.

For a deeper understanding, the experimental results were modeled by various kinetic models(Table 3S). The plots are expresses in Fig. 3Sa-d, and the data are collected in Table 2. PFO model gave a good correlation at 50 mg/L (R² = 0.992), but the calculated q_e_ (23.48 mg/g) deviated slightly from the experimental q_e_ (24.34 mg/g). At 100 mg/L, however, the PFO model showed a weaker fit (R² = 0.896) and overestimated q_e_ (78.98 mg/g) compared with the experimental value (44.07 mg/g). In contrast, the PSO model provided a strong fit for both concentrations, with higher correlation coefficients (R² = 0.998 at 50 mg/L and 0.950 at 100 mg/L). The calculated qe values (27.26 and 56.63 mg/g, respectively) were closer to the experimental results, suggesting that the CdS-QDs uptake byHMC@CH@CaAl-LDH is primarily dominated by chemisorption, where valence forces operate through electron exchange or sharing between the nanobiocomposite surface and CdS-QDs^61^. The intraparticle diffusion (IPD) model showed linear plots with relatively good correlations (R² = 0.922 and 0.980), indicating that diffusion into pores contributed to the overall adsorption process. However, the non-zero intercepts (C = 4.75 and 0.629) suggest that intraparticle diffusion was not the only rate-controlling step. Instead, the adsorption followed a multi-stage process, where boundary layer diffusion governed the rapid initial uptake, followed by slower diffusion into mesopores and interlayers^62^. Elovich model yielded high correlation coefficients (R² = 0.981 at 50 mg/L and 0.903 at 100 mg/L), confirming its suitability for describing the CdS-QDs adsorption process. The calculated parameter α, which represents the initial adsorption rate, was 18.32 and 18.27 mg/g.min at 50 and 100 mg/L, respectively. These relatively high values indicate the high affinity of HMC@CH@CaAl-LDH toward rapid binding with CdS-QDs at the beginning of the adsorption process^63^. The β values, which are related to the activation energy of chemisorption and surface heterogeneity, were 0.187 and 0.095 g/mg at the same initial concentrations. The lower β at higher concentration suggests that as more CdS-QDs accumulate on the surface, interactions become less favorable, and adsorption progresses more slowly due to site saturation and repulsive effects among adsorbed species^64^. This behavior is consistent with the hierarchical nature of the composite, where external functional groups of chitosan and surface sites of calcite besides LDH are rapidly occupied, leaving less accessible mesopores and interlayer domains for later stages. Overall, the kinetic analysis demonstrates that CdS-QDs uptake by HMC@CH@CaAl-LDH follows PSO mechanism, controlled by chemisorption with contributions from intraparticle diffusion. The rapid initial uptake and high adsorption capacity highlight the effectiveness of the hierarchical nanobiocomposite in capturing CdS-QDs from aqueous solutions.

**Table 2:** Kinetic models of CdS-QDs adsorption by HMC@CH@CaAl-LDH.

Kinetic model	Kinetic parameters	50 mg/L	100 mg/L
*Pseudo*-first order (PFO)	q_e(exp)_: (mg/g)	24.34	44.07
	q_e(calc)_: (mg/g)	23.48	78.98
	k_1_ : (min^-1^)	0.167	0.175
	R^2^	0.992	0.896
*Pseudo-*second order (PSO)	q_e(exp);_ (mg/g)	24.34	44.07
	q_e(calc);_ (mg/g)	27.26	56.63
	k_2_ : (g/mg.min)	0.012	0.003
	R^2^	0.998	0.950
Intraparticlediffusion (IPD)	K_id_: (mg/g. min^1/2^)	4.01	8.19
	C: (mg/g)	4.75	0.629
	R^2^	0.922	0.980
Elovich	α: (mg/g. min)	18.32	18.27
	β: (mg/g )	0.187	0.095
	R^2^	0.981	0.903

#### Influence of initial CdS-QDs concentration and isotherm modeling

Isotherm studies are important to investigate the adsorption mechanism and the adsorbate-adsorbent interactions. adsorption capacity of HMC@CH@CaAl-LDH was systematically assessed by diverse initial CdS-QDs concentrations, and the equilibrium data were subsequently modeled using five adsorption isotherm models(Table 3S): Langmuir, Freundlich, Dubinin–Radushkevich (D–R), Temkin, and Flory–Huggins (F–H), as illustrated in Fig. 4S(a–f). The variation of equilibrium adsorption capacity with equilibrium concentration (q_e_–C_e_ plot) is also shown in Fig. 5S. The corresponding linear equations and the derived parameters are presented in Table 3.At low initial CdS-QDs concentrations (20–50 mg/L), the adsorption capacity increased progressively, demonstrating a large number of accessible active sites and strong adsorbate–adsorbent interactions. As shown in Fig. 4Sa, as the concentration increased further, a slower rise in qₑ was observed, indicating the gradual saturation of active adsorption sites on HMC@CH@CaAl-LDH surface. This behavior is typical of the heterogeneous and hierarchical mesoporous architecture of the nanobiocomposite, which provides a highly accessible and interconnected pore network that enables efficient initial diffusion and binding of CdS-QDs, followed by progressive pore filling and increased site competition at higher concentrations^65^.

**Table 3:** Isotherm parameters of various models ofCdS-QDs adsorption by HMC@CH@CaAl-LDH.

Isotherm model	Isotherm parameters	
Langmuir	q_max_: (mg/g)	24.7
	b: (L/mg )	0.052
	R_L_	0.49–0.16
	R^2^	0.995
Freundlich	n	2.01
	K_F_(L/mg )	2.65
	R^2^	0.978
Dubinin-Radushkevich (D-R)	q_s_: (mg/g)	17.97
	K_ad_: (mol^2^/kj^2^ )	0.189
	E_s_: (kJ/ mol)	1.63
	R^2^	0.937
Temkin	a_T_: (L/g )	0.414
	b_T_: ( mg/L)	416.7
	B:( J/mol)	5.95
	R^2^	0.995
Flory-Huggins (F-H)	n	0.902
	K_FH_​​:(L/mg)	0.0485
	ΔG°: (kJ/mol)	-7.497
	R^2^	0.992

The adsorption mechanism was examined in details via several isotherm models to the equilibrium data. The Langmuir model showed an excellent fit (R² = 0.995), suggesting that adsorption mainly takes place as monolayer coverage on a uniform surface with finite binding sites. The maximum adsorptive capacity (q_max_) was calculated as 24.7 mg/g, which is consistent with the large surface area and hierarchical porosity of the composite. The dimensionless separation factor (R_L_ = 0.49–0.16) lies between 0 and 1, confirming the adsorption process is favorable across the studied concentration range^66^.The Freundlich model also fitted the data well (R² = 0.978), suggesting the presence of heterogeneous adsorption sites. The Freundlich constant (K_F_ = 2.65 L/mg) reflects a good adsorption capacity, while the value of *n* = 2.01 (> 1) indicates favorable adsorption intensity, consistent with a heterogeneous surface composed of chitosan functional groups, calcite pores, and LDH interlayers^67^.The D–R isotherm showed a lower correlation (R² = 0.937), with a saturation capacity (q_s_) of 17.97 mg/g. The mean adsorption energy (E_s_ = 1.63 kJ/mol) was below 8 kJ/mol, indicating that physisorption contributes to the overall process^68^. However, considering the superior fit of the Langmuir and PSO models, it is likely that the overall CdS-QDs adsorption onto HMC@CH@CaAl-LDH nanobiocomposite, involves both chemisorption at functional groups and physisorption within the mesopores and interlayers. The Temkin model also described the equilibrium data well (R² = 0.995). The Temkin constant B, referred to the calculated heat of adsorption as 5.95 J/mol, indicating moderate CdS-QDs and HMC@CH@CaAl-LDH interactions and reflecting the contributions from physisorption^69^. The fit to Temkin model suggests that adsorption energy decreases gradually with increasing surface coverage due to CdS-QDs repulsion, which is consistent with the trend observed at higher concentrations. Finally, the Flory–Huggins (F–H) model gave a high correlation (R² = 0.992), further supporting the feasibility of the process. The F-H equilibrium constant (K_FH_ = 0.0485 L/mg) and negative value of Gibbs free energy (ΔG° = − 7.497 kJ/mol), implying the spontaneous nature of CdS-QDs adsorption onto HMC@CH@CaAl-LDH. The value of n (0.902) suggests that a nearly one-to-one replacement of interlayers anions and water molecules by CdS-QDs occurs at adsorption sites^70^.

Taken together, the isotherm analysis demonstrates that CdS-QDs adsorption onto HMC@CH@CaAl-LDH is predominantly monolayer chemisorption on homogeneous sites (Langmuir), with contributions from multilayer adsorption on heterogeneous domains (Freundlich and Temkin) with spontaneous thermodynamic feasibility (Flory–Huggins).

#### Reusability of HMC@CH@CaAl-LDH nanobiocomposite

**Fig. 11:**
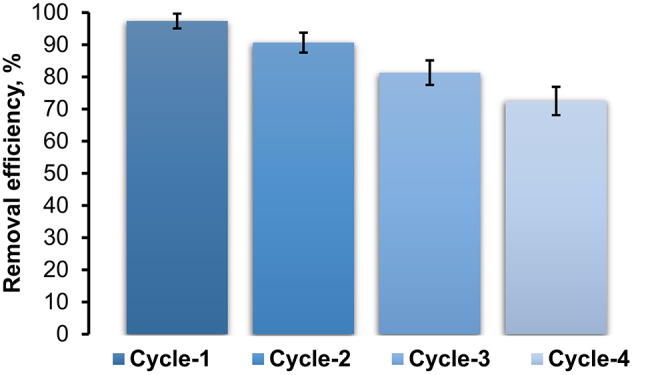
The removal efficiency of HMC@CH@CaAl-LDHtoward CdS-QDs after multiple cycles, at 25 °C, pH7 and 30 min contact time.

The reusability of HMC@CH@CaAl-LDH was also explored by four successive adsorption–desorption cycles to assess its potential for practical water treatment applications. Figure 11, demonstrates that the nanobiocomposite maintains high removal efficiency toward CdS-QDs even after multiple cycles. After the second and third cycles, the removal efficiency slightly decreased to 90.7% and 81.3%, respectively, showing only a marginal loss of performance. Even after the fourth cycle, the nanobiocomposite retained removal efficiency at 72.5%. The slight efficiency loss after repeated cycles can be attributed to partial blockage of active adsorption sites and incomplete desorption of CdS-QDs from inner pores. However, the successful structural preservation of the HMC@CH@Ca/Al-LDH nanobiocomposite after regeneration process was confirmed by FT-IR analysis. As shown in Fig. 6S, the regenerated nanobiocomposite retained the same characteristic spectral peaks as the fresh material.

#### Effect of ionic strength

The influence of ionic strength on the removal of CdS-QDs by HMC@CH@CaAl-LDH nanobiocomposite was evaluated in the presence of different concentrations of NaCl (0–500 mg/L) at an initial CdS-QDs concentration (100 mg/L) and pH 7. In the absence of NaCl, the nanobiocomposite exhibited a high CdS-QDs removal efficiency of 88.1%. As shown in Fig. 7S, upon increasing the NaCl concentration to 100 mg/L, the removal efficiency declined to 82.1%, followed by a further gradual decrease to 75.7% at 500 mg/L. The observed decrease in CdS-QDs removal efficiency with increasing ionic strength can be attributed primarily to electrostatic screening effects. In addition, the presence of Na⁺ and Cl⁻ ions may partially compete with CdS-QDs for charged or ion-exchangeable sites, minimizing the adsorption efficiency. Despite this, the nanobiocomposite maintained reasonable removal efficiency even at elevated ionic strength, suggesting that non-electrostatic interactions also play an important role in CdS-QDs adsorption. These include hydrogen bonding with hydroxyl and amino groups from chitosan, surface complexation with calcite functional groups, and physical entrapment within the hierarchical porous structure of HMC.

#### Application of HMC@CH@CaAl-LDH in CdS-QDs removal from polluted water samples

The potential applicability of the HMC@CH@CaAl-LDH nanobiocomposite in recovery of CdS-QDs from real environmental waters was evaluated using three different types of water samples: tap water, seawater, and industrial wastewater. Each water sample was spiked with a known concentration of CdS-QDs (10 mg/L) and treated under optimized conditions using 50 mg HMC@CH@CaAl-LDH. The results are summarized in Table 4.In tap water, the removal efficiency of CdS-QDs increased significantly over three consecutive treatment runs, starting at 87.6% in the first run, rising to 94.2% in the second, and reaching a maximum of 98.7% in the third. A similar improvement was observed for both seawater and industrial wastewater, where the removal efficiency increased from 78.2% to 81.9% in the first run to 93.7% and 96.4% in the third run, respectively. The slightly lower efficiencies observed in seawater and wastewater compared to tap water can be attributed to their higher ionic strength and the presence of competing ions and organic contaminants, which may initially hinder CdS-QDs adsorption by partially blocking the active sites and pores of the nanobiocomposite^71^. However, successive treatment cycles gradually overcome these effects as residual CdS-QDs are progressively removed. Overall, these results demonstrate the high efficiency, robustness, and versatility of the HMC@CH@CaAl-LDH nanobiocomposite for the remediation of CdS-QDs from different aqueous environments. Although the developed HMC@CH@Ca/Al-LDH nanobiocomposite demonstrated promising adsorption performance, the present study was conducted under batch laboratory conditions. Further investigations are required to evaluate its long-term stability, continuous-flow performance, and large-scale applicability under real industrial wastewater conditions.

**Table 4:** Application of HMC@CH@CaAl-LDH for CdS-QDs removal from contaminated water samples.

Water sample		Tap water	Seawater	Wastewater
% Removal	1st run	87.6	78.2	81.9
	2nd run	94.2	87.3	91.1
	3rd run	98.7	93.7	96.4

#### Adsorption mechanism of CdS-QDs

**Scheme 2:**
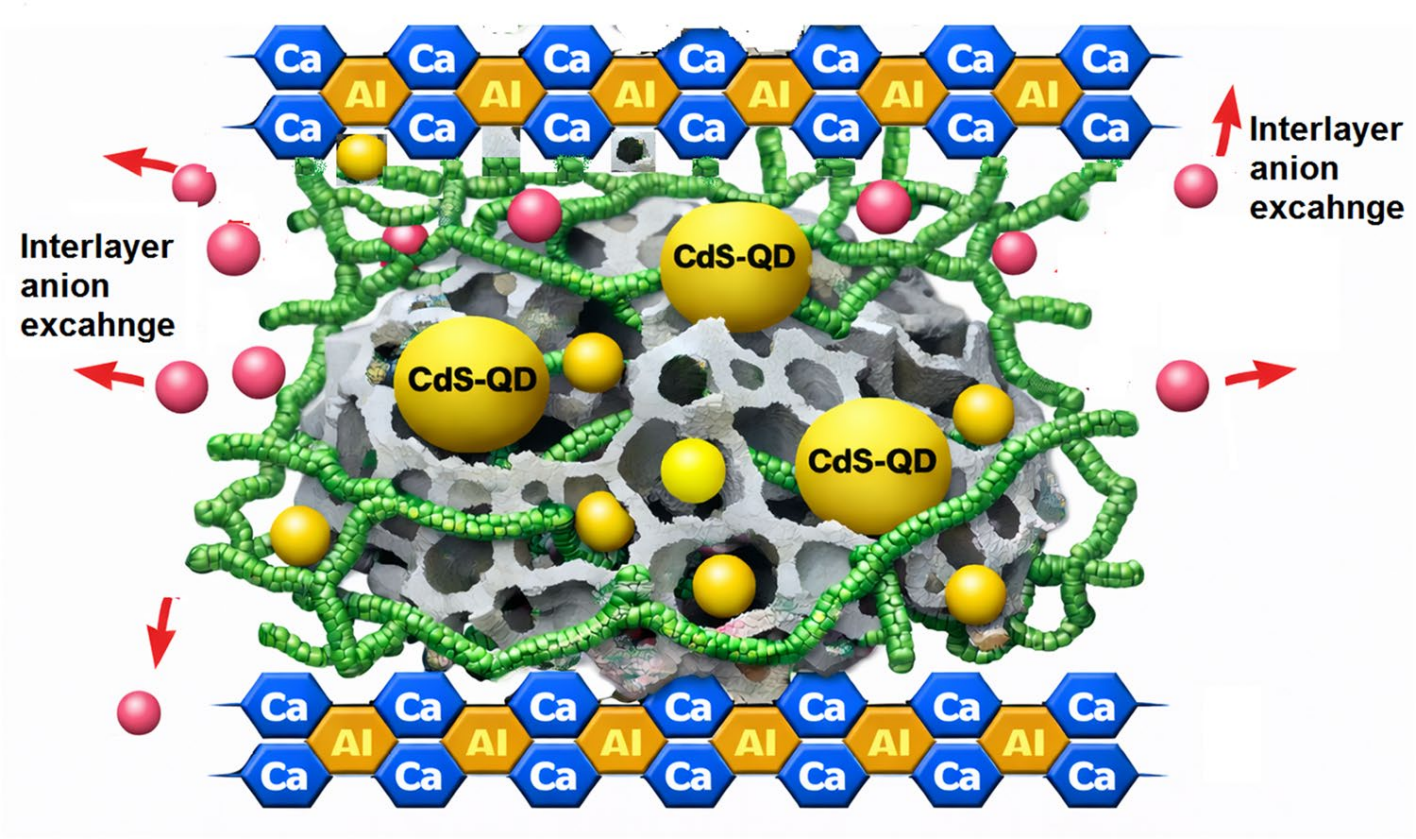
Graphical representation of CdS-QDs mechanistic removal by HMC@CH@CaAl-LDH nanobiocomposite.

The adsorption of CdS-QDs by HMC@CH@CaAl-LDH proceeds via a synergistic multi-mechanistic pathway, enabled by its porous hierarchical structure and multifunctional surface chemistry. The multi-stage process, described in Scheme 2, involves: (i) rapid chemisorption via coordination and hydrogen bonding at external functional groups, as confirmed by the pseudo-second-order kinetics, (ii) electrostatic attraction and anion exchange of CaAl-LDH interlayers, and (iii) slower intraparticle diffusion and physisorption within mesoporous and interlayer domains. Equilibrium isotherm analysis indicates predominant monolayer adsorption on homogeneous sites, with additional contributions from heterogeneous and physical interactions. These combined effects of chemisorption, anion exchange, and diffusion account for the high affinity and efficient removal of CdS-QDs by HMC@CH@CaAl-LDH from aqueous systems.

## Conclusion

A novel remediation of hazardous CdS-QDs, addressing their growing concern as emerging nanomaterial contaminants in water systems was achieved by a successfully synthesized nanobiocomposite. Hierarchical mesoporous calcite (HMC) and CaAl-LDH layers were integrated by chitosan hydrogel (CH) to produce a multifunctional HMC@CH@CaAl-LDH nanobiocomposite, exhibiting a high CdS-QDs removal efficiency of 97.4% and 88.1% at initial concentrations of 50 and 100 mg L^− 1^, respectively, under neutral pH. Adsorption kinetics followed the pseudo-second-order model, indicating chemisorption as the dominant uptake mechanism. Isotherm analyses confirmed monolayer adsorption on homogeneous sites (Langmuir), with contributions from heterogeneous domains (Freundlich, D–R, and Temkin). Thermodynamic parameters verified that the process was spontaneous and endothermic. Reusability tests demonstrated sustained performance over multiple cycles. Importantly, HMC@CH@CaAl-LDH nanobiocomposite performed effectively in recovery of CdS-QDs pollutant from real water samples. Overall, HMC@CH@CaAl-LDH represents a promising, sustainable, and versatile nanobiocomposite for addressing health risks associated with CdS-QDs in aquatic environments.

## Supplementary Information

Below is the link to the electronic supplementary material.

Supplementary Material 1

## Data Availability

All data generated or analyzed during this study are included in this published article and its supplementary information files.

## References

[1] Sulaiman, J. M. A. et al. Recent advances in carbon nanomaterials: Removal, photodegradation and electrochemical detection of tetracycline, a review. *Inorg. Chem. Commun.* **174**, 113897 (2025).

[2] Rosales, S. et al. Systematic review of carbon quantum dots (CQD): Definition, synthesis, applications and perspectives. *Renew. Sustain. Energy Rev.* **219**, 115854 (2025).

[3] Wu, M. et al. Molybdenum and tungsten dichalcogenides quantum dots: Properties, synthesis, and energy applications. *Coord. Chem. Rev.* **546**, 217043 (2026).

[4] Lei, W. et al. Photocatalytic degradation of methylene blue by CdS quantum dots biosynthesized by cysteine synthetase TtCsa1 from Tetrahymenathermophila. *Inter J. Biolog Macromol.* **305**, 141166 (2025). 10.1016/j.ijbiomac.2025.141166 39971067

[5] Campalani, C. & Monbaliu, J. C. M. Towards sustainable quantum dots: Regulatory framework, toxicity and emerging strategies. *Mater. Sci. Eng. R. Rep.* **163**, 100940 (2025).

[6] Pashootan, P. et al. Biomedical advances and clinical challenges of graphitic carbon nitride quantum dots: A comprehensive review. *Inorg. Chem. Commun.* **182**, 11563 (2025).

[7] Hu, L., Zhong, H. & He, Z. Toxicity evaluation of cadmium-containing quantum dots: A review of optimizing physicochemical properties to diminish toxicity. *Colloids Surf. B*. **200**, 111609 (2021). 10.1016/j.colsurfb.2021.111609 33588242

[8] Issa, N. A. et al. Incorporation of cadmium sulfide quantum dots in photoactive layer of quaternary organic solar cell. *Opt. Mater.* **159**, 116638 (2025).

[9] Lee, S. Y. et al. Lee, general guide for adsorption of cadmium sulfide (CdS) quantum dots by successive ionic layer adsorption and reaction (SILAR) for efficient CdS-sensitized photoelectrochemical cells. *Appl. Surf. Sci.* **589**, 152898 (2022).

[10] Rempel, S. V., Kuznetsova, Y. V., Ulitko, M. V. & Rempel, A. A. In vitro study of cytotoxicity of cadmium sulfide quantum dots in aqueous solutions. *Colloids Surf. A*. **710**, 136243 (2025).

[11] Sun, D. et al. Toxic effects and mechanistic insights of cadmium telluride quantum dots on the homeostasis and regeneration in planarians. *J. Hazard. Mater.* **486**, 137047 (2025). 39754879 10.1016/j.jhazmat.2024.137047

[12] Mimona, M. A. et al. Quantum dot nanomaterials: Empowering advances in optoelectronic devices. *Chem. Eng. J. Adv.* **21**, 100704 (2025).

[13] Kuznetsova, Y. V., Popov, I. D. & Gerasimov, E. .Rempel, Cadmium sulfide quantum dots in water media: Enhanced photoluminescence, dispersion and stability. *J. Mol. Liq*. **371**, 121084 (2023).

[14] Jamal, F. et al. Review of Metal Sulfide Nanostructures and their Applications. *ACS Appl. Nano Mater.* **6** (9) 7077–7106 (2023).

[15] He, W. et al. Metal-organic framework biocomposites based on proteins, polysaccharides, and nucleic acids: Synthesis, properties, and applications. *Coord. Chem. Rev.* **546**, 217052 (2026).

[16] Jalilian, M., He, Q. S. & Hu, Y. Synthesis of bimetallic biocomposite materials prepared from activated hydrochar/biochar for methylene blue removal via Fenton oxidation coupled with adsorption. *Chem. Eng. Sci.* **320**, 122514 (2026).

[17] Şenol, Z. M., Arslanoğlu, H., Keskin, Z. S., Mehmeti, V. & Messaoudi, N. E. Biosorption of rhodamine B and sunset yellow dyes on cross-linked chitosan-alginate biocomposite beads: Experimental and theoretical studies. *Inter J. Biolog Macromol.* **298**, 139264 (2025). 10.1016/j.ijbiomac.2024.139264 39824421

[18] Ahmad, S. et al. Al-Harthi, review of performance, mechanism, and challenges of layered double hydroxide-based biocomposites for the adsorptive removal of dye contaminants from water and wastewater. *J. Water Process. Eng.* **70**, 106837 (2025).

[19] Zhang, W. et al. The preparation of layered hierarchical and cube-shaped magnetic Fe3O4/CaCO3 for efficient enrichment of Pb(II) from aqueous solutions. *Environ. Nanotechnol. Monit. Manage.* **16**, 100600 (2021).

[20] Guerrero, J. D., Arias, E. R. & Gutierrez, L. B. Enhancing copper and lead adsorption in water by in-situ generation of calcium carbonate on alginate/chitosan biocomposite surfaces. *Inter J. Biolog Macromol.* **266**, 131110 (2024). 10.1016/j.ijbiomac.2024.131110 38522694

[21] Xuan, Y., Feng, X., Liu, S. & Liu, X. Layered double hydroxide-based membranes for advanced water treatment: Structural engineering and multifunctional applications. *Chem. Eng. J.* **511**, 161746 (2025).

[22] Liu, G. et al. Magnetic Fe_3_O_4_/NiFe Layered Double Hydroxide Composites for Rapid and Efficient Nitrate Removal from Aqueous Solutions. *J. Alloys Comp.* 183467. (2025).

[23] Farhan, A. et al. Muhammad Bilal Asif ^i^Progress in layered double hydroxides (LDHs): Synthesis and application in adsorption, catalysis and photoreduction. *Sci. Total Environ.* **912**, 169160 (2024). 38086474 10.1016/j.scitotenv.2023.169160

[24] Iftekhar, S., Srivastava, V., Ramasamy, D. L., Naseer, W. A. & Sillanpää, M. A novel approach for synthesis of exfoliated biopolymeric-LDH hybrid nanocomposites via in-stiu coprecipitation with gum Arabic: Application towards REEs recovery. *Chem. Eng. J.* **347**, 398–406 (2018).

[25] Iftekhar, S., Srivastava, V., Hammouda, S. B. & Sillanpää, M. Fabrication of novel metal ion imprinted xanthan gum-layered double hydroxide nanocomposite for adsorption of rare earth elements. *Carbohydr. Polym.* **194**, 274–284 (2018). 29801840 10.1016/j.carbpol.2018.04.054

[26] Bessaies, H. et al. Synthesis of novel adsorbent by intercalation of biopolymer in LDH for the removal of arsenic from synthetic and natural water. *J. Environ. Sci.* **91**, 246–261 (2020). 10.1016/j.jes.2020.01.028 32172974

[27] Jiang, J. et al. Hierarchical CaCO_3_ particles self-assembled from metastable vaterite and stable calcite during the decomposition of Ca(HCO_3_)_2_. *Cryst. Eng. Comm.* **19** (48), 7332–7338 (2017).

[28] Zhang, X., Wang, Y., He, J. & Long, F. Hierarchical CaCO3 assembled by porous nanorods with excellent performance of Cd (II) adsorption in waste water. *J. Water Process. Eng.* **68**, 106528 (2024).

[29] Zhu, Y., Shan, S., Hu, T., He, L. & Zhou, H. Hierarchical pore carbon-calcium nanocages for highly effective removal of ammonium-nitrogen and phosphorus. *Fuel Process. Technol.* **247**, 107804 (2023).

[30] Yin, W. et al. G.–T. Zhou. Removal and recovery of silver nanoparticles by hierarchical mesoporous calcite: Performance, mechanism, and sustainable application. *Environ. Res.* **187**, 109699 (2020). 32480024 10.1016/j.envres.2020.109699

[31] Zhang, J. et al. Uncovering the role of carboxyl anchor groups in promoting resonance energy transfer from CdS quantum dots to molecular acceptors. *J. Luminesc*. **286**, 121406 (2025).

[32] Azhari, N. J. et al. Nano calcite-templated hierarchical ZSM-5 synthesized via solvent-free route for selective production of rich-aromatic, high-octane green gasoline. *Fuel* **381**, 133345 (2025).

[33] Al-Assy, W. H., Mostafa, M. M., Elfeky, S. M. & Abozeid, S. M. Synergistic enhancement of antibacterial, anticancer, and antioxidant activities of Novel Co(II) and Cu(II) complexes loaded into Ca/Al-layered double hydroxide nanosheets. *J. Mol. Struct.* **1349**, 143646 (2026).

[34] Siddiq, H. A. et al. El-Bindary, Efficient removal of tetracycline by VCo-layered double hydroxide encapsulated with chitosan: Optimization via Box-Behnken design, and thermodynamics. *Inter J. Biolog Macromol.* **296**, 139565 (2025). 10.1016/j.ijbiomac.2025.139565 39798768

[35] Sakr, M. E. M. et al. Fluorescence and photostability studies of a Xanthenone-based dye via CdS quantum dot complexation. *Phys. B: Condens. Matter*. **720**, 417996 (2026).

[36] Chen, J., Gao, P., Liu, J. & Zhu, Y. Flotation separation scheelite from calcite by using a novel depressant of Poly(sodium 4-styrenesulfonate). *Adv. Powder Technol.* **35** (11), 104664 (2024).

[37] Yang, Y. et al. Mechanochemical synthesis of Ca-Al-La layered double hydroxide for efficient soluble phosphorus immobilization in phosphogypsum. *Environ. Res.* **285**, 122447 (2025). 40716603 10.1016/j.envres.2025.122447

[38] Zhang, Y. et al. Highly efficient removal of Mn(II) and organic pollutant by adipate functionalized Ca-Al layered double hydroxides: Capture behavior and mechanism. *Colloids Surf. A*. **717**, 136774 (2025).

[39] Bansal, M. & Pal, B. Enhanced elimination of nitrate and nitrite ions from ground and surface wastewater using chitosan sphere-modified Mg-Al layered double hydroxide composite. *J. Ind. Eng. Chem.* **142**, 635–650 (2025).

[40] Yu, J. et al. MOF-derived Co-Ni layered double hydroxides/polyethyleneimine modified chitosan micro-nanoreactor for high-efficiency capture of uranium from seawater. *Carbohydr. Polym.* **323**, 121426 (2024). 37940255 10.1016/j.carbpol.2023.121426

[41] Tajat, N. et al. Synthesis of eco-friendly CaCO_3_@Zn-Al MMO core-shell nanoflowersphotocatalyst using bio–eggshell waste for improved photocatalytic degradation of RhB under visible light irradiation. *Environ. Res.* **263**, 120218 (2024). 39448007 10.1016/j.envres.2024.120218

[42] Li, L., Yang, Y., Lv, Y., Yin, P. & Lei, T. Porous calcite CaCO3 microspheres: Preparation, characterization and release behavior as doxorubicin carrier. *Colloids Surf. B*. **186**, 110720 (2020). 10.1016/j.colsurfb.2019.110720 31855688

[43] Ouyang, E. et al. Chitin/calcite composite extracted from shell waste as a low-cost adsorbent for removal of tetracycline and ciprofloxacin: Effects and mechanisms. *Chemosphere* **353**, 141503 (2024). 38382718 10.1016/j.chemosphere.2024.141503

[44] Hajibeygi, M., Oghan, M. & Mobaraki, A. Ca–Al LDH modified with sulfonated Fe_3_O_4_@SiO_2_ core-shell as an efficient reinforcement for the preparation of magnetic poly(amide-imide) nanocomposites. *Mater. Today Chem.* **48**, 103004 (2025).

[45] Arafa, E. G. et al. Eco-friendly and biodegradable sodium alginate/quaternized chitosan hydrogel for controlled release of urea and its antimicrobial activity. *Carbohydr. Polym.* **291**, 119555 (2022). 35698383 10.1016/j.carbpol.2022.119555

[46] Ahmad, S. et al. A review of performance, mechanism, and challenges of layered double hydroxide-based biocomposites for the adsorptive removal of dye contaminants from water and wastewater. *J. Water Process. Eng.* **70**, 106837 (2025).

[47] Baldovino-Medrano, V. G., Niño-Celis, V., Isaacs, R. & Giraldo Systematic Analysis of the Nitrogen Adsorption–Desorption Isotherms Recorded for a Series of Materials Based on Microporous–Mesoporous Amorphous Aluminosilicates Using Classical Methods. *J. Chem. Eng. Data*. **68** (9), 2512–2528 (2023).

[48] Adim, S. et al. Green four-component reaction and dye adsorption studies of new ZnCoAl-LDH and ZnCoCr-LDH materials. *J. Organomet. Chem.* **1036**, 123719 (2025).

[49] Renukadevi, S., Manimozhi, V., Sivakumar, E. K. T. & Jaisankar, V. Pet-coke derived GO/Chitosan polymer composite: Preparation, characterization, and biomedical evaluation. *J. Ind. Chem. Soc.* **102** (7), 101760 (2025).

[50] Liu, R. et al. Innovative amorphous calcium carbonate for superior anionic dye adsorption towards near-zero discharge. *Sep. Purif. Technol.* **361**, 131349 (2025).

[51] Reyad, A. et al. Layered double hydroxide/conductive polymer nanocomposites: Toward multifunctional biocompatible nanoadsorbents for wastewater treatment applications. *Sep. Sci. Technol.* **59** (15), 1294–1311 (2024).

[52] Yardımcı, B. & Kanmaz, N. An effective-green strategy of methylene blue adsorption: Sustainable and low-cost waste cinnamon bark biomass enhanced via MnO_2_. *J. Environ. Chem. Eng.* **11**, 110254 (2023).

[53] Mülazımoğlu, E., Yardımcı, B. & Kanmaz, N. Cross-linked chitosan and iron-based metal-organic framework decoration on waste cellulosic biomass for pharmaceutical pollutant removal, Proc. Safe. Environ. Prot. 196 106948. (2025).

[54] Yardımcı, B. & Kanmaz, N. Ecosafe-design of carboxymethyl cellulose encapsulated polyphenolic bio-nanocomposite valorized for sustainable industrial textile dye removal. *J. Environ. Chem. Eng.* **13**, 115321 (2025).

[55] Huang, Z. et al. Facile synthesis of a MOF-derived magnetic CoAl-LDH@chitosan composite for Pb (II) and Cr (VI) adsorption. *Chem. Eng. J.* **449**, 137722 (2022).

[56] Priya, V. N., Rajkumar, M., Mobika, J. & Sibi, S. P. L. Adsorption of As (V) ions from aqueous solution by carboxymethyl cellulose incorporated layered double hydroxide/reduced graphene oxide nanocomposites: Isotherm and kinetic studies. *Environ. Technol. Innov.* **26**, 102268 (2022).

[57] Zhang, J. W. et al. Multiple pollutants removal by carbon sphere and layered double hydroxide composites: Adsorption behavior and mechanisms. *J. Environ. Chem. Eng.* **10** (3), 108014 (2022).

[58] Mahmoud, S. E. M. E., Abdel-Fattah, T. M., Mahmoud, M. E. & Díaz, E. Efficient removal performance of polystyrene microplastics from strongly acidic solutions by two functionalized nanosized biochars derived from low-cost sustainable sources. *Sci. Total Environ.* **969**, 178892 (2025). 40020576 10.1016/j.scitotenv.2025.178892

[59] Mahmoud, M. E., Abdelwahab, M. S. & Ibrahim, G. A. A. Surface functionalization of magnetic graphene oxide@bentonite with α-amylase enzyme as a novel bionanosorbent for effective removal of Rhodamine B and Auramine O dyes. *Mater. Chem. Phys.* **301**, 127638 (2023).

[60] Zubair, M., Ihsanullah, I., Abdul Aziz, H., Azmier Ahmad, M. & Al-Harthi, M. A. Sustainable wastewater treatment by biochar/layered double hydroxide composites: Progress, challenges, and outlook. *Bioresour. Technol.* **319**, 124128 (2021). 32979597 10.1016/j.biortech.2020.124128

[61] Yang, J. et al. Hierarchical porous N-doped carbon encapsulated CoFe2O4-CoO nanoparticles derived from layered double hydroxide/chitosan biocomposite for the enhanced degradation of tetracycline. *Sep. Purif. Technol.* **295**, 121291 (2022).

[62] Rodríguez-González, M. A. et al. Kinetic study of Pb(II) adsorption in polluted waters from tannic materials. *J. Water Process. Eng.* **75**, 108042 (2025).

[63] Wang, J. & Guo, X. Rethinking of the intraparticle diffusion adsorption kinetics model: Interpretation, solving methods and applications. *Chemosphere* **309**, 136732 (2022). 36223824 10.1016/j.chemosphere.2022.136732

[64] Eslami, A. & Juibari, N. M. Potentially toxic elements removal from aqueous solution using green synthesized ZnCo_2_O_4_ nanoparticles: a comparative study of adsorption isotherm and kinetic using linear and non-linear models. *J. Ind. Chem. Soc.* **102** (9), 101907 (2025).

[65] Lopresto, C. G., Gentile, M., Caravella, A., Candamano, S. & Calabrò, V. De-acidification of waste cooking oils by adsorption on industrial waste: Kinetic analysis of a green pretreatment for biodiesel production. *Chemosphere* **380**, 144460 (2025). 40334615 10.1016/j.chemosphere.2025.144460

[66] Abdulhameed, A. S., Al Omari, R. H., Younes, M. K. & Algburi, S. Carboxylated chitosan-phthalate/ZrO2 nanocomposite for removal of methylene blue dye: Characterization and adsorption modeling via response surface methodology. *J. Mol. Struct.* **1339**, 142386 (2025).

[67] Althobaiti, S. A., Nabil, G. M. & Mahmoud, M. E. Insight into optimization of doxorubicin removal by a novel nanobiocomposite of doped molybdenum carbide and zinc ferrite onto pomegranate peels nanobiochar (Mo₂C-ZnFe₂O₄@PPNB). *J. Mol. Liq*. **429**, 127597 (2025).

[68] Siddiq, H. A. et al. El-Bindary, Efficient removal of tetracycline by VCo-layered double hydroxide encapsulated with chitosan: Optimization via Box-Behnken design, and thermodynamics. *Inter J. Biolog. Macromol.* **296**, 139565 (2025). 10.1016/j.ijbiomac.2025.139565 39798768

[69] Allou, N. B., Atheba, P., Saikia, J. & A.-A.Soro, K. Ello. Methylparaben adsorption on calcined layered double hydroxides: Kinetics and isotherm modeling. *Chem. Data Collect.* **57**, 101187 (2025).

[70] Alshammari, N. A. H. et al. Synthesis of pomegranate peel-activated carbon encapsulated onto carboxymethylcellulose and polyethylenimine for cadmium (II) adsorption: Optimization, kinetics and isotherm modeling. *Int. J. Biol. Macromol.* **310**, 143348 (2025). 40262686 10.1016/j.ijbiomac.2025.143348

[71] Chu, K. H., Hashim, M. A., Hayder, G. & Bollinger, J. C. Rooting out faulty adsorption models. Comment on Magnetic Prussian blue nanoshells are controllable (sic) anchored on the surface of molybdenum disulfide nanosheets for efficient separation of radioactive cesium from water. *Sci. Total Environ.* **921**, 171118 (2024). 38382619 10.1016/j.scitotenv.2024.171118

