# Supplementary material for: Removal of CdS-QDs pollutant from wastewater by interconnected Ca/Al layered double hydroxides with hierarchical mesoporous calcite and chitosan hydrogel
Source: Sci Rep. 2026 Apr 2;16:11363. doi: 10.1038/s41598-026-43797-x (PMC13049060; doi:10.1038/s41598-026-43797-x)
Supplement: Supplementary file 1 — Supplementary Material 1 [file 41598_2026_43797_MOESM1_ESM.docx]

**Adsorptive capture of cadmium sulfide quantum dots pollutant from wastewater by interconnected calcium aluminum layered double hydroxides with hierarchical mesoporous calcite and chitosan hydrogel**

**Mohamed E. Mahmoud*, Mohamed F. Amira, Enass A.I. Saleh, Hany Abdel-Aal**

Faculty of Sciences, Chemistry Department, Alexandria University, Alexandria, Egypt. Email: memahmoud10@yahoo.com

**Table 1S.**Specifications of chemicals used in this study

| **Reagent** | **Chemical Formula** | **F.W (g/mol)** | **Assay** | **Company** |
| --- | --- | --- | --- | --- |
| Chitosan  from shrimp shells | (C_6_H_11_O_4_N)_n_ |  | ≥75.0% | Sigma–Aldrich Chemical Company, St Louis, USA |
| Sodium sulfide hydrate | Na_2_S· xH_2_O | 78.04 (anhydrous basis) | ≥60.0% |  |
| Calcium nitrate tetrahydrate | Ca(NO₃)₂·4H₂O | 236.15 | ≥99.0% |  |
| Aluminium nitrate nonahydrate | Al(NO₃)₃·9H₂O | 375.13 | ≥ 99.9% |  |
| Sodium nitrate | NaNO_3_ | 84.995 | ≥99.0% |  |
| Hydrochloric acid | HCl | 36.46 | ≥37.0% | Oxford Lab Fine Chem LLP, India |
| Acetic acid | C_2_H_4_O_2_ | 60.05 | ≥99.0% |  |
| Sodium hydroxide | NaOH | 39.99 | ≥97.0 % |  |
| Ethanol | C_2_H_6_O | 46.07 | ≥ 99.5% |  |
| Disodium ethylenediaminetetraacetate dihydrate | C_10_H_14_N_2_Na_2_O_8_.2H_2_O | 372.24 | ≥99.0% |  |
| Cadmium sulfate hydrate | 3CdSO₄·8H₂O | 769.54 | ≥ 98.0% |  |
| Glutaraldehyde | C_5_H_8_O_2_ | 100.12 | ≥ 50.0% |  |
| Calcium chloride hexahydrate | CaCl_2_.6H_2_O | 219.08 | ≥98.0% |  |
| Sodium Carbonate | Na_2_CO_3_ | 105.99 | ≥99.5% |  |

**Table 2S.** Specifications of instrumental techniques used in this study

| **Instrumental technique** | **Model** | **Conditions** |
| --- | --- | --- |
| **FT-IR** | BRUKER VERTEX  70 Fourier Transform  infrared spectrophotometer | In the range 400–4500 cm−1 |
| **SEM** | Scanning electron  microscopic JSM6360LA, JEOL Ltd | A carbon tap was used as a substrate for SEM measurements using an ion sputtering coating device (JEOL-JFC-1100E) |
| **EDX** | Energy Dispersive X-ray  JSM-lT200, JEOL  Ltd | A carbon tap was used as a substrate for EDX measurements. Acceleration voltage 20.00 kV, WD 10.00 mm, Live time 30.00, high vacuum mode |
| **TEM** | Transmission electron  microscopy,model  JEOL JEM-2100F,  Japan | Acquiring the images at 80 to 200 kV |
| **XRD** | The X-ray diffraction by XRD Shimadzu lab X6100, Japan | The XRD generator worked at 40 kV,  30 mA, and λ = 1 Å utilizing target Cu-Kα with secondary monochromatic.2-Theta wa  s started at 10° and ended at 80°.The diffraction data was recorded with step of 0.02° and a time of 0.6 s at room temperature |
| **Thermal**  **Gravimetric Analysis (TGA)** | Perkin-Elmer TGA7 Thermobalance or by Linseis STAPT1000 using a temperature from 20 to 700 |  |
| **BET** | Brunauer–Emmett–  Teller by BELSORP-mini II, BEL J | The BET surface areas by nitrogen  adsorption–desorption isotherms were determined for 24 h |
| **UV/Vis**  **spectrophotometer** | Ultraviolet/visible  spectrophotometer by  V-530 JASCO | UV/ViS spectrophotometer in between the range of wavelength from 190 nm to 1100 nm was used in the absorption measurement |
| **pH-meter** | Adwa pH-meter | Standard buffers 4.01, 7.00 and 10.00 were utilized in the calibration of Adwa pH-meter which used in the measurements of solutions pH |

**Table 3S.** Thermodynamic, kinetic and isotherm models

| **Van’t Hoff equations** | ${\Delta G}^{\boldsymbol{^{\circ}}}=-\mathrm{RT}\ln K$  $\Delta G\boldsymbol{^{\circ}}=\Delta H\boldsymbol{^{\circ}}-T\Delta S\boldsymbol{^{\circ}}$ | | **R** : the universal gas constant  (8.314 J/ mol. K)  **T** : the absolute temperature (K)  **K** : the equilibrium constant. | |
| --- | --- | --- | --- | --- |
| *Pseudo*-first order (PFO) | | $\ln\left( \boldsymbol{q}_{\boldsymbol{e}}\boldsymbol{-}\boldsymbol{q}_{\boldsymbol{t}} \right)\boldsymbol{=}\boldsymbol{ln}\boldsymbol{q}_{\boldsymbol{e}}\boldsymbol{-}\boldsymbol{k}_{\boldsymbol{1}}\boldsymbol{t}$ | | k_1_ : (min^-1^), PFO rate constant |
| *Pseudo-*second order (PSO) | | $\frac{\boldsymbol{t}}{\boldsymbol{q}_{\boldsymbol{t}}}\boldsymbol{=}\frac{\boldsymbol{1}}{\boldsymbol{k}_{\boldsymbol{2}}\mathbf{q}_{\mathbf{e}}^{\mathbf{2}}}\mathbf{+}\frac{\mathbf{t}}{\mathbf{q}_{\mathbf{e}}}$ | | k_2_ : (g mg^-1^ min^-1^), PSO rate constant |
| Intraparticlediffusion (IPD) | | $\boldsymbol{q}_{\boldsymbol{t}}\mathbf{=}\boldsymbol{k}_{\boldsymbol{id}}\boldsymbol{t}^{\mathbf{1}\mathbf{/}\mathbf{2}}\mathbf{+}\boldsymbol{C}$ | | K_id_: (mg. g^-1^ min^-1/2^), intraparticle diffusion rate constant  C: (mg g^-1^), thickness of the boundary layer |
| Elovich | | $\boldsymbol{q}_{\boldsymbol{t}}\mathbf{=}\frac{\mathbf{1}}{\boldsymbol{\beta}}\ln\mathbf{(}\boldsymbol{\alpha\beta}\mathbf{)}\mathbf{+}\frac{\mathbf{1}}{\boldsymbol{\beta}}\ln\mathbf{t}$ | | α: (mg g^-1^ min^-1^), surface coverage for the initial rate of adsorption  β: (mg g^-1^ ), the activation energy of chemisorption |
| **Langmuir** | | $\frac{\boldsymbol{C}_{\boldsymbol{e}}}{\boldsymbol{q}_{\boldsymbol{e}}}\boldsymbol{=}\frac{\boldsymbol{1}}{{\boldsymbol{b}\boldsymbol{.}\boldsymbol{q}}_{\boldsymbol{max}}}\boldsymbol{+}\frac{\boldsymbol{C}_{\boldsymbol{e}}}{\boldsymbol{q}_{\boldsymbol{max}}}$  $\boldsymbol{R}_{\boldsymbol{L}}\boldsymbol{=}\boldsymbol{1}\boldsymbol{/}\boldsymbol{(}\boldsymbol{1}\boldsymbol{+}\boldsymbol{b}\boldsymbol{.}\boldsymbol{C}_{\boldsymbol{o}}\mathbf{)}$ | | **q_max_ :** (mg g^-1^), maximum adsorption capacity  **b**: (L mg^-1^ ), Langmuir constants  **R_L_** : Separation factor |
| **Freundlich** | | $\boldsymbol{ln}\boldsymbol{q}_{\boldsymbol{e}}\boldsymbol{=}\boldsymbol{ln}\boldsymbol{K}_{\boldsymbol{F}}\boldsymbol{+}\frac{\boldsymbol{1}}{\boldsymbol{n}}\boldsymbol{ln}\boldsymbol{C}_{\boldsymbol{e}}$ | | **n** : intensity of adsorbent  **K_F_ :**  (L mg^-1^ ), Freundlich constant |
| **Dubinin-Radushkevich (D-R)** | | $\boldsymbol{ln}\boldsymbol{q}_{\boldsymbol{e}}\boldsymbol{=}\boldsymbol{ln}\boldsymbol{q}_{\boldsymbol{s}}\boldsymbol{-}\boldsymbol{(}\boldsymbol{K}_{\boldsymbol{ad}}\mathbf{Ɛ}^{\boldsymbol{2}}\boldsymbol{)}$  $\mathbf{Ɛ}\boldsymbol{=}\boldsymbol{RTln}\boldsymbol{(}\boldsymbol{1}\boldsymbol{+}\frac{\boldsymbol{1}}{\boldsymbol{C}_{\boldsymbol{e}}}\boldsymbol{)}$  $\boldsymbol{E}_{\boldsymbol{s}}\boldsymbol{=}\frac{\boldsymbol{1}}{\sqrt{{\boldsymbol{2}\boldsymbol{K}}_{\boldsymbol{ad}}}}$ | | **q_s_ :** (mg g^-1^), Saturation adsorption capacity  **K_ad_:**  (mol^2^ kj^-2^ ), D-R constant  **E_s_ :** (kJ mol⁻¹), energy of adsorption |
| **Temkin** | | $\boldsymbol{q}_{\boldsymbol{e}}\boldsymbol{=}\frac{\boldsymbol{RT}}{\boldsymbol{b}_{\boldsymbol{T}}}\boldsymbol{ln}\boldsymbol{a}_{\boldsymbol{T}}\boldsymbol{+}\frac{\boldsymbol{RT}}{\boldsymbol{b}_{\boldsymbol{T}}}\boldsymbol{ln}\boldsymbol{C}_{\boldsymbol{e}}$  $\boldsymbol{q}_{\boldsymbol{e}}\boldsymbol{=}\boldsymbol{Bln}\boldsymbol{a}_{\boldsymbol{T}}\boldsymbol{+}\boldsymbol{Bln}\boldsymbol{C}_{\boldsymbol{e}}$  $\boldsymbol{B}\boldsymbol{=}\frac{\boldsymbol{RT}}{\boldsymbol{b}_{\boldsymbol{T}}}$ | | **a_T_:**  (L g^-1^ ), Temkin isotherm equilibrium binding constant  **b_T_:** ( mg L^-1^), Temkin isotherm adsorption constant  **B :**( J mol^-1^), the adsorption heat constant |
| **Flory-Huggins (F-H)** | | $\boldsymbol{ln}\frac{\boldsymbol{\theta}}{\boldsymbol{C}_{\boldsymbol{e}}}\boldsymbol{=}\boldsymbol{ln}\boldsymbol{K}_{\boldsymbol{FH}}\boldsymbol{+}\boldsymbol{nln}\boldsymbol{(}\boldsymbol{1}\boldsymbol{-}\boldsymbol{\theta}\boldsymbol{)}$  Where, ***θ***(q_e_/q_max_) is the degree of surface coverage, ${\boldsymbol{\Delta}\mathbf{G}}^{\boldsymbol{^{\circ}}}\mathbf{=}\mathbf{-}\mathbf{RT}\ln\mathbf{K}_{\mathbf{HF}}$ | | **𝑛:** the number of adsorbates occupying adsorption sites  ***K_FH_***​:(L mg^-1^), F-H equilibrium constant |

**Fig. 1S.** UV-Vis absorption spectrum of CdS-QDs

**Fig. 2S.** Van’t Hoff plot of CdS-QDs adsorption by HMC@CH@CaAl-LDH, at pH7 and 30 min contact time.

|  |  |
| --- | --- |
|  |  |

**Fig. 3S.** (a) Pseudo-first order, (b) Pseudo-second order, (c) Intraparticle diffusion and (d) Elovich kinetic models of CdS-QDs adsorption by HMC@CH@CaAl-LDH

|  |  |
| --- | --- |
|  |  |
|  |  |

**Fig. 4S.** (a) Effect of CdS-QDs concentration on adsorption capacity, (b) Langmuir, (c) Freundlich, (d) Dubinin-Radushkevich, (e) Temkin and (f) Flory-Huggins isotherm models

**Fig. 5S.** The adsorption capacity (qe) variation vs equilibrium concentration (Ce) of CdS-QDs at 25 °C, pH7 and 30 min contact time.


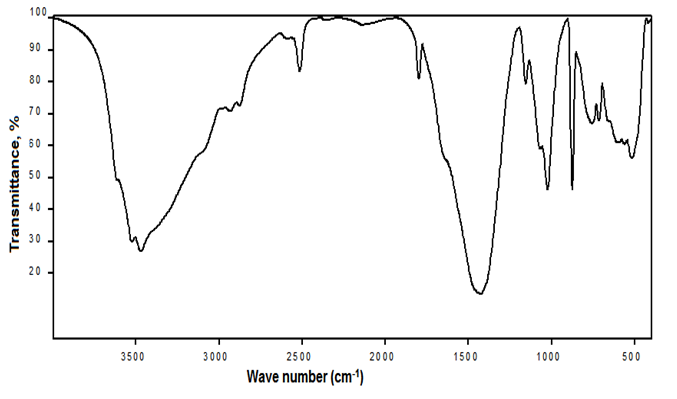


**Fig. 6S.** FT-IR spectrum of regenerated HMC@CH@CaAl-LDH

**Fig. 7S.** Effect of ionic strength on removal efficiency of CdS-QDs, at pH7
